# Genetic variation in *CCDC93* is associated with elevated central systolic blood pressure, impaired arterial relaxation, and mitochondrial dysfunction

**DOI:** 10.1371/journal.pgen.1011151

**Published:** 2024-09-09

**Authors:** Nitin Kumar, Min-Lee Yang, Pengfei Sun, Kristina L. Hunker, Jianping Li, Jia Jia, Fangfang Fan, Jinghua Wang, Xianjia Ning, Wei Gao, Ming Xu, Jifeng Zhang, Lin Chang, Y. Eugene Chen, Yong Huo, Yan Zhang, Santhi K. Ganesh

**Affiliations:** 1 Division of Cardiovascular Medicine, Department of Internal Medicine, University of Michigan Medical School, Ann Arbor, Michigan, United States of America; 2 Department of Human Genetics, University of Michigan Medical School, Ann Arbor, Michigan, United States of America; 3 Department of Computational Medicine and Bioinformatics, University of Michigan Medical School, Ann Arbor, Michigan, United States of America; 4 Department of Cardiology, Peking University First hospital, Beijing, China; 5 Department of Cardiology, Tianjin Medical University General Hospital, Tianjin, China; 6 Laboratory of Epidemiology, Tianjin Neurological Institute, Tianjin, China; 7 Department of Neurology, Tianjin Medical University General Hospital, Tianjin, China; 8 Department of Cardiology, Peking University Third hospital, Beijing, China; 9 Institute of Cardiovascular Disease, Peking University First Hospital, Beijing, China; 10 Hypertension Precision Diagnosis and Treatment Research Center, Peking University First Hospital, Beijing, China; Case Western Reserve University School of Medicine, UNITED STATES OF AMERICA

## Abstract

Genetic studies of blood pressure (BP) traits to date have been performed on conventional measures by brachial cuff sphygmomanometer for systolic BP (SBP) and diastolic BP, integrating several physiologic occurrences. Genetic associations with central SBP (cSBP) have not been well-studied. Genetic discovery studies of BP have been most often performed in European-ancestry samples. Here, we investigated genetic associations with cSBP in a Chinese population and functionally validated the impact of a novel associated coiled-coil domain containing 93 (*CCDC93*) gene on BP regulation. An exome-wide association study (EWAS) was performed using a mixed linear model of non-invasive cSBP and peripheral BP traits in a Han Chinese population (N = 5,954) from Beijing, China genotyped with a customized Illumina ExomeChip array. We identified four SNP-trait associations with three SNPs, including two novel associations (rs2165468-SBP and rs33975708-cSBP). rs33975708 is a coding variant in the *CCDC93* gene, c.535C>T, p.Arg179Cys (MAF = 0.15%), and was associated with increased cSBP (β = 29.3 mmHg, *P* = 1.23x10^-7^). CRISPR/Cas9 genome editing was used to model the effect of *Ccdc93* loss in mice. Homozygous *Ccdc93* deletion was lethal prior to day 10.5 of embryonic development. *Ccdc93*^+/-^ heterozygous mice were viable and morphologically normal, with 1.3-fold lower aortic Ccdc93 protein expression (*P* = 0.0041) and elevated SBP as compared to littermate *Ccdc93*^+/+^ controls (110±8 mmHg vs 125±10 mmHg, *P* = 0.016). Wire myography of *Ccdc93*^+/-^ aortae showed impaired acetylcholine-induced relaxation and enhanced phenylephrine-induced contraction. RNA-Seq transcriptome analysis of *Ccdc93*^+/-^ mouse thoracic aortae identified significantly enriched pathways altered in fatty acid metabolism and mitochondrial metabolism. Plasma free fatty acid levels were elevated in *Ccdc93*^+/-^ mice (96±7mM vs 124±13mM, *P* = 0.0031) and aortic mitochondrial dysfunction was observed through aberrant Parkin and Nix protein expression. Together, our genetic and functional studies support a novel role of *CCDC93* in the regulation of BP through its effects on vascular mitochondrial function and endothelial function.

## Author summary

More than 1000 loci have been reported in previous gene discovery efforts for blood pressure and hypertension. cSBP is a unique blood pressure trait that has not been as extensively studied as peripheral BP, and correlates of cSBP may provide a better indication of future cardiovascular events. In this study we replicated several BP associations in a Han Chinese cohort and found a novel association with a coding variant in *CCDC93* (rs33975708, c.535C>T, p.Arg179Cys, MAF 0.15%) which was strongly associated with higher cSBP. To model loss of *Ccdc93* and functionally validate the human genetic association, a new transgenic mouse was created. *Ccdc93* homozygous deletion was embryonic lethal, and mice with heterozygous loss of *Ccdc93* exhibited impaired arterial relaxation and enhanced contractile responses. Aortic RNA-seq analysis of *Ccdc93*^*+/-*^ heterozygous mice identified metabolic dysregulation and mitochondrial dysfunction, which was validated by features of elevated plasma free fatty acids and increased aortic Parkin and Nix protein expression. Improved knowledge of the genes involved in BP regulation is needed to develop effective therapies to manage elevated BP, and this study identified the gene *CCDC93* as a novel regulator of blood pressure and vascular function.

## Introduction

Elevated blood pressure (BP) is the leading modifiable risk factor for cardiovascular disease and stroke [1,2]. BP is influenced by genetic, demographic, and environmental factors, and their interactions. Previous genome-wide association studies (GWAS) have identified and validated variants at more than 1000 loci with modest effects on population BP [3–6], explaining in aggregate ~6% of the trait variance [7]. Differences according to ancestry in the genetic architecture of BP and hypertension in the population have been identified [8] and highlight the importance of genetic studies in diverse ancestry populations [9].

Conventionally, BP is measured at the brachial artery by automatic BP monitors or auscultation of Korotkoff sounds. Thus, measured BP is an integrated readout of both the forward wave of BP according to cardiac output and arterial elasticity as well as a reflected wave mediated by total peripheral vascular resistance [10]. Although central systolic BP (cSBP) and peripheral SBP (pSBP) may share similar mean values, there are significant differences between these two measures. cSBP is determined by the interactions between the left ventricle and the vasculature. cSBP is elevated due to increased forward and reflected wave amplitudes and earlier return of the reflected wave to the proximal aorta and it is greatly influenced by arterial stiffness [11,12]. Additionally, BP amplification from the central aorta to peripheral arteries occurs. These factors together lead to discrepancies between central BP and peripheral BP, with underlying physiologic etiologies that are not defined through conventional BP measurement [13–15]. Although peripheral BP is strongly associated with central BP [16], cSBP responds differently to certain BP-lowering drugs [17] and cSBP may be more closely correlated with future cardiovascular events as compared to peripheral BP measured at the brachial artery [18,19], as well as measures of related cardiovascular disease risks [12,17,19–26].

cSBP is heritable, with estimated heritability 0.18 in a twin study [27]; conventionally measured SBP and diastolic BP (DBP) has estimated heritability 0.17–0.52 [28]. Despite numerous GWAS of peripheral BP, few studies have focused on genetic associations with central BP [29,30]. Furthermore, most BP GWAS studies have been conducted in European ancestry samples, and relatively fewer have been conducted in East Asian samples [31].

In the current study, we performed an exome-wide association study (EWAS) using the Illumina ExomeChip genotyping array for discovery associations with cSBP, pSBP, pDBP, and peripheral mean arterial pressure (pMAP) in Han Chinese populations, followed by replication experiments. We generated a novel transgenic mouse based upon the EWAS findings with heterozygous loss of *Ccdc93* and conducted functional validation experiments.

## Results

### PUUMA cohort discovery study samples

The PUUMA Study included 5,954 subjects with peripheral BP measurements (pSBP, pDBP, and pMAP) and 4,938 subjects with cSBP. In the discovery PUUMA cohort, 63.3% (n = 3,769) of participants were female. Average cSBP was 133.1±18.6 mmHg and average pSBP was 133.7±16.7 mmHg. Other traits and covariates are summarized in **Table 1**.

**Table 1:** Characteristics of participants in the discovery stage EWAS.

Characteristics	N (%) or Mean±S.d.
N	5954
Women	3769 (63.3)
Age	56.9±9.0
Body mass index	26.1±3.4
Systolic blood pressure	133.7±16.7
Diastolic blood pressure	75.0±10.0
Mean arterial pressure	94.6±10.8
Central systolic blood pressure	133.1±18.6
Anti-hypertensive medication	1890 (31.9)

### PUUMA cohort discovery stage EWAS

**Fig 1:**
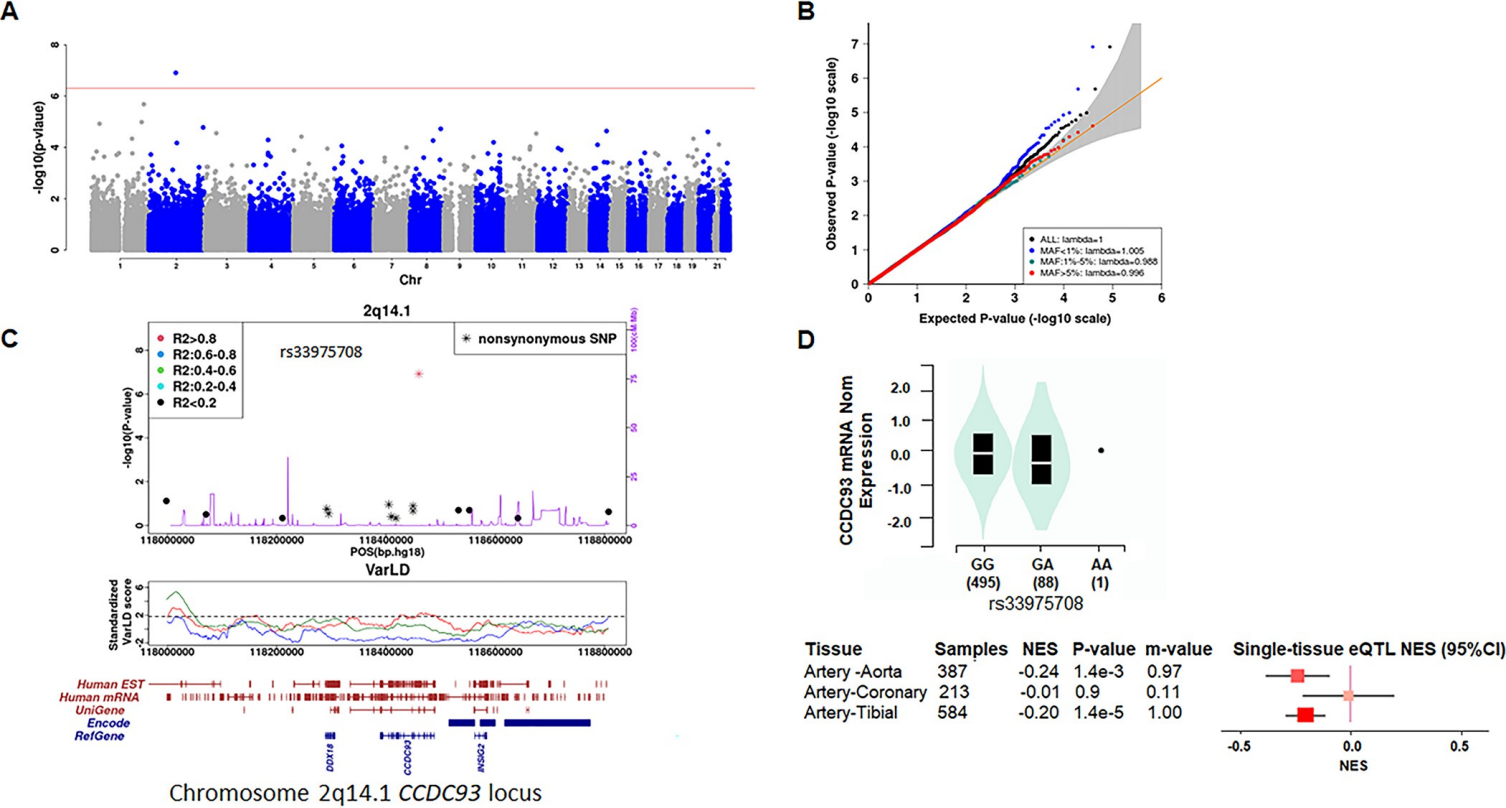
Exome-wide association study (EWAS) for central systolic blood pressure (cSBP) and eQTL plot. ExomeChip SNPs meeting quality control were analyzed in the EWAS. Results are shown in a **(A)** Manhattan plot and **(B)** Quantile-Quantile plot; λ_GC_ value was 1.0. **(C)** Regional association plots with gene annotation for the chromosome 2q14.1 region associated with cSBP is shown with the index SNP rs33975708 and additional SNPs within 500 kbp in each direction. Top panel: Regional cSBP association plot for the *CCDC93* variant. LD (linkage disequilibrium) was calculated from our samples. Non-synonymous variants were annotated using ANNOVAR. The genetic recombination rate is based on Hapmap release 22. Middle Panel: Standardized varLD scores illustrate LD variations between populations (CEU vs. JPT+CHB, CEU vs. YRI, YRI vs. JPT+CHB) using genome positions from the Hapmap 3 reference. The red line represents the comparison between CEU (European ancestry) and JPT+CHB (East Asian ancestry), the purple line indicates CEU versus YRI (African ancestry), and the green line represents YRI versus JPT+CHB. Bottom Panel: Gene/transcript annotations are sourced from the reference downloaded from the UCSC database (EST, mRNA, uniGene, Encode, and RefGene). **(D)** eQTL plots for rs33975708 (chr2_117986054_G_A_b38 eQTL) and ENSG00000125633.10 (*CCDC93*), for human arterial tissues in GTEx data. Significant eQTLs were queried from the GTEx V8 portal for arterial tissues, which showed that the tibial artery had significant eQTL for rs33975708 and *CCDC93* gene, based on GTEx defined permutation-based multiple test correction significance threshold. The eQTL violin plot demonstrated that the risk allele or alternative allele, A, for rs33975708, decreases the expression of *CCDC93*. A cross-tissue meta-analysis of 49 GTEx tissues using METASOFT tested the posterior probability that an eQTL effect exists in each tissue was also reported (meta-analysis RE2 P-value: 1.96E-15). A large m-value (>0.9) indicated the tissue was predicted to have an eQTL effect, whereas a small m-value (<0.1) indicated that it was predicted not to have an eQTL effect. Both the tibial artery and aorta artery had a larger m-value>0.9, indicating a strong eQTL effect. The risk allele of rs33975708, A, was associated with lower expression of *CCDC93*, in all the three arterial tissues (NES<0).

We conducted an EWAS for each BP trait, using mixed linear models, adjusted for age, age^2^, BMI, and sex, with resulting λ_GC_ values ranging from 0.997 to 1.008. The Manhattan plot, quantile-quantile plot (QQ), and regional association plot for cSBP are shown in **Fig 1A–1C**. Results for pSBP, pDBP and pMAP are shown in **S1–S3 Figs**. We identified four locus-trait combinations that reached the Bonferroni corrected genome wide significance threshold of *P*-value<5.8 × 10^−7^ in the discovery analysis, including 2 novel SNP-trait associations and 2 previously reported associations (**Table 2**).

**Table 2:** EWAS SNP-trait associations. SNP-trait associations meeting genome-wide significance are shown.

Trait	SNP	Locus	Chr	Position^1^	A1	A2	MAF	β	SE	*P*	Gene
pSBP	rs2165468	p15.2	10	3516105	A	C	0.458	1.77	0.34	1.55x10^-07^	*PITRM1*(dist = 301072), *KLF6*(dist = 302083)
pDBP	rs13149993	q21.21	4	81158545	A	G	0.423	1.06	0.21	3.16x10^-07^	*PRDM8*(dist = 33063), *FGF5*(dist = 29197)
pMAP	rs13149993	q21.21	4	81158545	A	G	0.423	1.24	0.24	1.67x10^-07^	*PRDM8*(dist = 33063), *FGF5*(dist = 29197)
cSBP	rs33975708	q14.1	2	118743630	A	G	0.002	29.31	5.53	1.23x10^-07^	*CCDC93*

^1^Assembly version: GRCh37 (hg19)

The associations of rs13149993 with pDBP and rs13149993 with pMAP reported in previous studies [32] were replicated in our East-Asian population. rs13149993 on chromosome 4q21.21 locus within *FGF5* was associated with pDBP (risk allele: A; risk allele frequency: 0.42; β: 1.06; SE: 0.21; *P*: 3.16×10^−7^) and pMAP (risk allele: A; risk allele frequency: 0.42; β: 1.24, SE: 0.24; *P*: 1.67×10^−7^). The two novel locus-trait combinations we identified were rs2165468 located on chromosome 10p15.2 and between *PITRM1* (dist = 301,072 bp from GRCh37 genome assembly) and *KLF6*, was associated with pSBP (risk allele: A; risk allele frequency: 0.46; β = 1.77; SE = 0.34; *P* = 1.55×10^−7^), and rs33975708 located on chromosome 2q14.1 in *CCDC93* was associated with cSBP (risk allele: A; risk allele frequency = 0.002; β = 29.31; SE = 5.53; *P* = 1.23×10^−7^).

To confirm major published BP association signals in our cohort, the association of a genetic risk score (GRS) based upon published SBP and DBP GWAS results, which included more than one million samples from the International Consortium for Blood Pressure (ICBP) and UK Biobank (UKB) cohorts, and the corresponding BP traits in our BP EWAS cohort samples were performed. GRS were weighted using effect sizes from the published GWAS of the same BP trait (5). Though subjects of the current study and previously published studies differed in ancestry, significant associations were identified with SBP (GRS-association of 611 SNPs β = 0.68, SE = 0.06, *P* = 3.42×10^−30^) and DBP (GRS-association of 734 SNPs β = 0.59, SE = 0.06, *P* = 2.5×10^−24^) in UKB with adjustment for age, age^2^, gender, BMI, and the top 10 PCs, and in ICBP (**S1 Table**).

### Replication of novel SNP-trait associations

We conducted replication analyses of the 2 novel SNP-trait associations from the PUUMA EWAS. Given the rarity of rs33975708 (MAF in East Asians: 0.15%) and the lack of available cohorts with existing cSBP measurement, as well as that BP measurements may not be static across time for each sample, we attempted replication in two manners. First, we brought back subjects for an additional visit and collected an additional cSBP measurement. From our discovery stage cohort, 3448 individuals (84% samples of the discovery stage subjects) were evaluated at a mean follow-up of 2.35±0.08 years after the first visit and BP measurements. The association with rs33975708 for subjects with both first-visit and second-visit measurements (N = 2897), utilizing only the second visit cSBP measurement was significant, with a similar effect size as the discovery stage association of rs33975708 and cSBP (β = 23.63, SE = 7.84, *P* = 2.58×10^−3^). In a secondary analysis of the union set of samples (N = 4938) from these two visits, cSBP was averaged across the visits to yield a long-term averaged cSBP (LTA-cSBP) value [33]. LTA-cSBP was associated with rs33975708, with a stronger effect estimate and lower p-value (β = 24.63, SE = 6.62, *P* = 9.95×10^−8^) as compared to the initial discovered association result (**S2 Table**). An independent replication analysis of rs33975708 with cSBP measured by the same technique was performed in the Ji cohort (N = 76) [34], although underpowered for detection of association, with 9 individuals carrying the rs33975708-A *CCDC93* risk allele and 67 individuals who did not have this risk allele; the association result was not statistically significant, but the effect direction was consistent with the discovery stage result (β = 11.18, SE = 9.64, *P* = 0.25). Inspection of the cSBP values showed that individuals in the Ji cohort with the rs33975708-A *CCDC93* risk allele had a mean cSBP of 175.1 mmHg (s.d. = 30.7 mmHg) whereas individuals who did not carry the risk allele had a mean cSBP of 162.5 mmHg (s.d. = 26.2 mmHg) with t-test *P*-value = 0.19. Meta-analysis of discovery stage and Ji County data showed consistent directions of effect: β = 24.82, SE = 4.80, *P* = 2.29x10^-7^ utilizing inverse-variance-weighted method, and *P* = 7.03x10^-8^ by employing a weighted sum of Z-scores method [35]. We tested replication of the association of rs2165468 with pSBP using two independent resources (Peking University First Hospital and Third Hospital). This association was replicated in 565 participants from Peking University First Hospital (β = 2.45, SE = 1.15, *P* = 0.034), but not replicated in 127 participants from third hospital (β = 1.7, SE = 2.38, *P* = 0.48), although under-powered analyses; in the meta-analysis of the two replication results, a positive association with consistent effect direction was observed (β = 2.3, SE = 1.04, *P* = 0.026) (**S2 Table**).

In PUUMA, *CCDC93*-rs33975708 was evaluated for pleiotropic associations with peripheral BP traits, pulse wave velocity and blood lipid levels, as major risk factors for atherosclerotic coronary artery disease. rs33975708 was significantly associated with several BP traits including pSBP and Ba-PWV. (**Table 3 and S4 Fig)**.

**Table 3:** Pleiotropy analysis of rs33975708. For participants taking anti-hypertensive medications, pSBP and pDBP measurements were adjusted +15 mmHg and +10 mmHg, respectively. For blood lipid, participants taking lipid-lowering drugs were excluded.

Trait	N	Sex and age adjusted model	
		β(95%CI)	*P*
**pSBP**			
GG	5936	ref	
AG	18	14.47(5.87,23.07)	0.001
**pDBP**			
GG	5936	ref	
AG	18	6.55(1.37,11.74)	0.013
**pPP**			
GG	5936	ref	
AG	18	7.92(2.09,13.74)	0.008
**pMAP**			
GG	5936	ref	
AG	18	9.19(3.27,15.11)	0.002
**Ba-PWV**			
GG	5654	ref	
AG	18	285.94(143.44,428.44)	8.51x10^-5^
**LDL cholesterol**			
GG	5295	ref	
AG	15	0.41(0.01,0.82)	0.047
**HDL cholesterol**			
GG	5296	ref	
AG	15	-0.11(-0.32,0.11)	0.328
**Total cholesterol**			
GG	5294	ref	
AG	15	0.37(-0.36,1.09)	0.318
**Triglycerides**			
GG	5308	ref	
AG	15	0.24(-0.35,0.84)	0.425

### Generation and characteristics of *Ccdc93* mutant mice and eQTL analysis

Given the challenges of replicating the effect of a low-frequency variant, we hypothesized that gene loss of function in a mouse deficient in *Ccdc93* expression would demonstrate higher BP and be a new model for functional studies of this effect. Supporting this direction, rs33975708 showed an expression QTL association with lower *CCDC93* expression in tibial artery in the GTEx database (*P* = 0.000014, **Fig 1D**), with the caveat that most GTEx tissues were from European ancestry individuals. In GTEx, eQTLs for each tissue have been precalculated through linear regression employing programs like fastQTL or MatrixQTL. GTEx calculated permutation-based empirical p-values and multiple test corrections to assess the significance of variant-gene pairs. After multiple test correction (Storey’s q value), we identified a significant association between rs33975708 and the cis-eQTL gene *CCDC93*, in tibial artery (*P* = 1.4x10-5, normalized effect size = -0.2, **Fig 1D**).

Using GTEx v8 data, we identified 275 expression-associated SNPs (eSNPs) for *CCDC93* in the aorta and 270 eSNPs in the tibial artery. Of these, 92 eSNPs were in linkage disequilibrium (LD) with rs33975708 (R2>0.2, +/- 500Kb) as per 1000G CEU population. All 92 proxy eSNPs showed the same effect as rs33975708 and *CCDC93*, with the minor allele linked to reduced *CCDC93* expression, indirectly supporting our study’s signals. Neither the specific SNPs nor any proxy SNP, when available, identified any eQTL associations for the additional variants, rs2165468-pSBP and rs13149993-pDBP/pMAP, using the updated GTEx v8 portal and other eQTL databases (such as the Human Genetic Variation Database). Only rs33975708-cSBP exhibited a significant association with *CCDC93* in tibial artery in GTEx v8.

**Fig 2:**
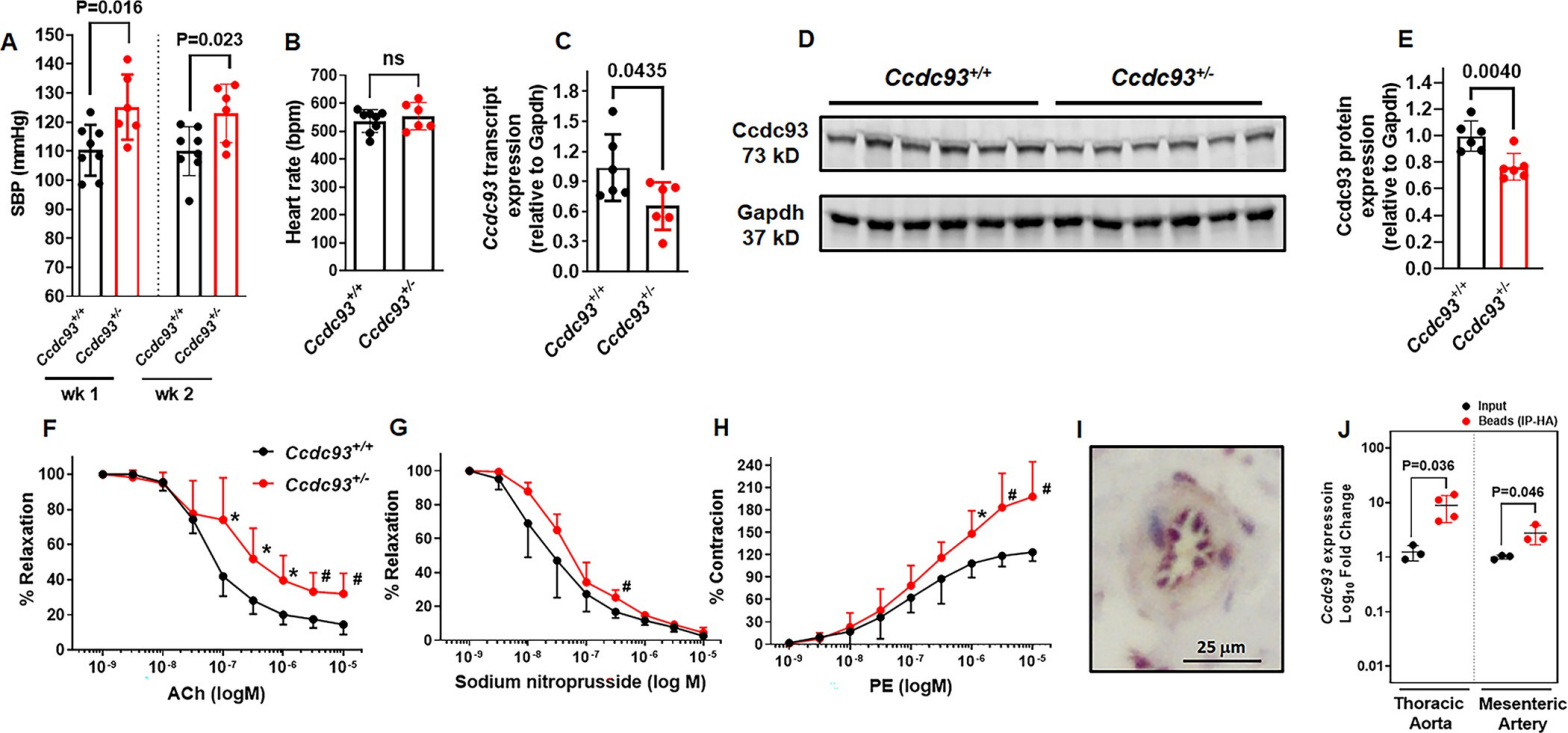
*Ccdc93*^*+/-*^ influences systolic blood pressure and arterial relaxation in mice. **(A)** *Ccdc93*^*+/-*^ mice displayed higher systolic blood pressure as compared to *Ccdc93*^*+/+*^ littermate controls. **(B)** Heart rate (bpm, beats per minute) was not different in *Ccdc93*^*+/-*^ mice as compared to *Ccdc93*^*+/+*^ littermate controls. Both male and female mice (N = 3–4 per genotype) were used in our study and since we identified no sex-specific signals (**S11 Fig**), further results are reported for the combined-sex analyses of all mice studied. **(C)** *Ccdc93* transcript expression and protein expression **(D-E)** were significantly lower in the descending thoracic aortae in *Ccdc93*^*+/-*^ mice as compared to the *Ccdc93*^*+/+*^ littermate control mice (N = 6–8 in each group). **(F-G)** In the descending thoracic aortae, *Ccdc93*^*+/-*^ (red) displayed significant impairment in acetylcholine (ACh)-induced endothelial relaxation (endothelial-dependent) and a mild impairment in sodium nitroprusside-induced relaxation (endothelium-independent) as compared to the littermate controls (black). Dose response curve of Ach and sodium nitroprusside were performed in aortic rings precontracted with phenylephrine (10 μM). **(H)** Phenylephrine (PE)-induced contraction was significantly higher in *Ccdc93*^*+/-*^ as compared to the littermate controls (*Ccdc93*^*+/+*^ N = 5, *Ccdc93*^*+/-*^ N = 6). The dose-response curve of PE is expressed as % maximal response to high KCl buffer (60 mM). Data were analyzed by two-sided unpaired t-test. *P*<0.05 was defined to be statistically significant (* *P*<0.05, # *P*<0.03). **(I)** Immunohistochemistry showed Ccdc93 protein expression was enriched in the endothelium of mesenteric artery of *Ccdc93*^*+/+*^ wild-type mouse (129/Sv). **(J)** Endothelial cell-specific translating ribosome affinity purification (EC-TRAP) confirmed that *Ccdc93* transcript expression was indeed enriched in the endothelial cells (immunoprecipitated-hemagglutinin IP-HA fraction) of thoracic aortae and mesenteric arteries of C57BL/6 wild-type mice (N = 3–4 in each group). All data are expressed as mean±s.d.

Using CRISPR/Cas9 genome editing in mouse embryos, we created a novel transgenic mouse to model heterozygous deletion of *Ccdc93*. *Ccdc93* heterozygous (*Ccdc93*^*+/-*^) mice have a 21-nucleotide deletion in a region spanning intron 6/exon 7 (2 nucleotides in intron 6 and 19 nucleotides in exon 7, **S5 Fig**), and significantly reduced aortic *Ccdc93* mRNA transcript and protein expression (**Fig 2C–2E**). *Ccdc93*^+/-^ mice were viable and morphologically normal. In contrast, *Ccdc93* null (*Ccdc93*^-/-^) mice were embryonic lethal and died prior to embryonic day E10.5 (**S6 Fig**), supporting that *Ccdc93* is indispensable for embryonic development. Timed mating was performed in *Ccdc93*^*+/-*^ X *Ccdc93*^*+/-*^ mice, and pregnant females were euthanized at E10.5 to assess embryonic abnormality in *Ccdc93*^*-/-*^. 5 fetoplacental units (FPUs) of total 56 were *Ccdc93*^-/-^ (9%, **S6A Fig**). Histological analysis at E10.5 of a single uterine horn revealed 2 resorption sites and 6 healthy FPUs. FPUs of *Ccdc93*^*+/-*^ heterozygote contained developing embryos that appeared viable and had no evidence of necrosis, inflammation, or hemorrhage (**S6B Fig**). Resorption sites of *Ccdc93*^*-/-*^ homozygous FPUs contained hemorrhage and necrotic debris accompanied by neutrophilic and lymphocytic inflammation within and around chorioallantoic membranes on the fetal side. There were no embryos or embryonic tissues present (**S6C Fig**), suggesting that *Ccdc93*^-/-^ embryos either did not develop or died very early in gestation.

Total body weight, and lean mass measured by echo magnetic resonance imaging (Echo MRI) were higher in *Ccdc93*^*+/-*^ heterozygous mice as compared to the littermate controls (**S12A Fig**). Heart weight, kidney weight and spleen weight normalized over body weight were not significantly different in *Ccdc93*^+/-^ as compared to littermate controls (**S7A–S7C Fig**). *Ccdc93*^+/-^ heterozygous mice displayed normal aortic histology on H&E and picrosirius red staining (**S8 Fig**) and normal peripheral blood counts (**S3 Table**).

### Mice deficient in *Ccdc93* demonstrated higher SBP and impaired arterial relaxation

Baseline SBP was significantly higher in *Ccdc93*^+/-^ mice measured at two time-points one week apart (week 1: 125.15±10.24 mmHg, week 2: 123.01±9.19 mmHg, **Fig 2A**) as compared to littermate *Ccdc93*^+/+^ mice (week 1: 110.31±8.17 mmHg, *P* = 0.016, week 2: 110.03±8.87 mmHg, *P* = 0.023). Heart rate was similar in *Ccdc93*^+/-^ and *Ccdc93*^+/+^ littermate control mice (**Fig 2B**). Both male and female mice (N = 3–4 per genotype) were studied, and secondary analyses were performed stratified by sex that identified no sex-specific signals (**S11 Fig**). Further results are reported for the combined-sex analyses of all mice studied. Vascular reactivity of aortae was evaluated *ex vivo* using wire myography. Descending thoracic aortic rings of *Ccdc93*^+/-^ showed impaired acetylcholine (ACh)-induced relaxation starting at ACh dose 10^−7^ M (*P* = 0.043) and relaxation remained impaired for all increasing ACh doses tested up to a maximal ACh response, corresponding to 86% vs 68% (*Ccdc93*^*+/+*^ vs *Ccdc93*^*+/-*^, *P* = 0.029 at Ach 10^−5^ M, **Fig 2F**). Endothelium-independent relaxation induced by sodium nitroprusside was impaired in *Ccdc93*^+/-^ aortic rings as compared to *Ccdc93*^+/+^ control aortic rings at 3.16×10^−7^ M sodium nitroprusside (*P* = 0.013, **Fig 2G**), although to an overall milder degree as compared to ACh-induced arterial relaxation.

Phenylephrine (PE)-induced contractile responses of *Ccdc93*^+/-^ aorta to a high concentration of PE starting at 10^−6^ M was significantly higher in *Ccdc93*^+/-^ when compared with *Ccdc93*^+/+^ littermate controls (*P* = 0.048) and remained highly contractile at all increasing PE doses, as compared to *Ccdc93*^+/+^ controls (maximal PE response at 10^−5^ M was 123% vs 198% normalized to maximal response to high KCl buffer (*Ccdc93*^*+/+*^ vs *Ccdc93*^*+/-*^, *P* = 0.015 **Fig 2H**).

### Arterial transcript expression studies to localize *Ccdc93* expression and identify mechanisms of *Ccdc93* deficiency

Based upon the findings of endothelial dysfunction in *Ccdc93*^*+/-*^ mice, we hypothesized that vascular *Ccdc93* expression is enriched in the endothelium. We thus evaluated endothelial cell expression of Ccdc93 protein in a wild-type mouse (129/Sv) by immunohistochemistry which demonstrated endothelial expression in the mesenteric artery (**Fig 2I**). Quantification of Ccdc93 protein expression showed 1.3 fold higher expression in the endothelium as compared to the non-endothelial cells in mouse aorta (**S15 Fig**); however due to high dispersion and some degree of background staining, we employed cell-type targeted mRNA quantification by performing endothelial cell-specific translating ribosome affinity purification (EC-TRAP) in thoracic aortae and mesenteric arteries of *Rpl22*^*fl/fl*^, *Tek-*^*Cre+/0*^ C57BL/6 wild-type mice, which showed that *Ccdc93* transcript expression was in fact enriched in endothelial cells (**Fig 2J**). As positive controls for the experiment, the expected enrichment of EC-specific transcript expression (*Tek*, *Cdh5* and *Vwf*) and depletion of SMC transcripts (*Tagln*, *Acta2* and *Cnn1*) were observed (**S9A and S9B Fig**) as well as immunostaining of EC-specific marker platelet and endothelial cell adhesion molecule-1 (PECAM-1) that showed expected staining of ECs in mouse aorta (**S16A and S16B Fig**). Immunostaining of PECAM-1 and Ccdc93 showed strong brown staining in the positive control (antibody present), which was not observed in the negative control of mouse aortic tissues (antibody absent) (**S16A–S16D Fig**). We queried the mouse and human single-cell RNA-seq database at Tabula Muris and GTEx respectively, which demonstrated *Ccdc93* expression in endothelial cells (**S14A–S14C Fig**).

**Fig 3:**
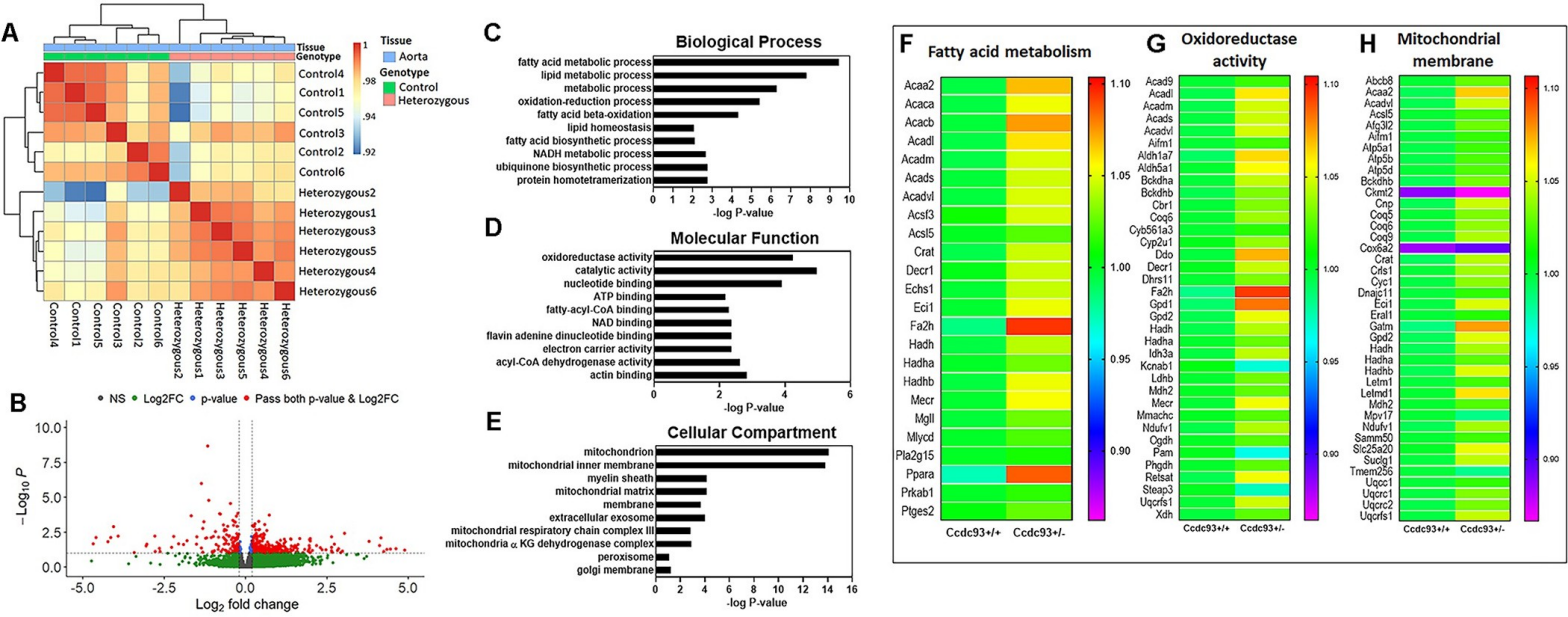
RNA-seq analysis of *Ccdc93*^*+/-*^ mouse aortae. Descending thoracic aortae of male and female *Ccdc93*^*+/+*^ littermate controls and *Ccdc93*^*+/-*^ heterozygous mice (N = 3 in each group) were analyzed by bulk RNA-Seq analysis. **(A**) A heat map represents the correlation between the samples of top differentially expressed genes by the genotype. Correlations were calculated using the Pearson correlation coefficient from the log-transformed raw reads counting per gene. **(B)** Volcano plot showing significantly upregulated genes (368) and downregulated genes (131) in thoracic aortae of *Ccdc93*^*+/-*^ compared to *Ccdc93*^*+/+*^ mice. **(C-E)** Gene ontology (GO) analysis of the differentially expressed genes of descending thoracic aortae of *Ccdc93*^*+/-*^ mice as compared to the littermate controls. GO is classified based on the **(C)** biological process, **(D)** molecular function and **(E)** cellular compartment and plotted against Benjamini adjusted P-value on the x-axis. **(F-H)** Significantly altered gene expression of fatty acid metabolic process, oxidoreductase activity and mitochondrial membrane affiliated gene regulators, are represented by heat maps. The normalized rlog transformed counts were used to generate heat maps (FDR<0.1).

To gain insights into the underlying molecular mechanisms of impaired arterial relaxation and high SBP in *Ccdc93*^+/-^ using unbiased transcriptome analysis, thoracic aortic RNA transcript expression was evaluated by bulk RNA-Seq. A heat map (**Fig 3A)** showed correlation between the samples of top differentially expressed genes according to genotype. PCA plot (**S10A Fig**) demonstrated no clear clustering of transcriptomes by mouse genotype, which may be partly explained by allelic compensation and high degree of global correlation in transcription expression profile between heterozygous and controls. Differential expression analysis comparing *Ccdc93*^+/-^ to *Ccdc93*^+/+^ littermate control aortae identified 368 significantly upregulated and 131 downregulated transcripts (FDR<0.1, log2FC>0.5, **Fig 3B**), with reduced aortic *Ccdc93* transcript expression observed as expected in *Ccdc93*^*+/-*^ mice, consistent with qPCR data (**S10B Fig**). Gene ontology and pathway analyses showed a strong enrichment of pathways of mitochondrial function including fatty acid metabolism, oxidoreductase pathway, mitochondrial fatty acid beta oxidation, and reactive oxygen species production (**Figs 3C–3H and S10C**). Transcripts involved in the mitochondrial reactive oxygen species (ROS) cascade including malate dehydrogenase 2 (*Mdh2*), xanthine dehydrogenase (*Xdh*), cytochrome c1 (*Cyc1*), aldehyde dehydrogenase 5 family member A1 (*Aldh5a1*), and aldehyde dehydrogenase family 1, subfamily A7 (*Aldh1a7*) were significantly higher in *Ccdc93*^*+/-*^ as compared to controls. *Cyc1* and *Aldh5a1*, *Aldh1a7* are elevated under oxidative stress and function as ROS scavenger and detoxification of highly reactive aldehydes, respectively [36,37]. Acetyl-Coenzyme A acyltransferase 2 (*Acaa2*), Acyl coenzyme-A dehydrogenase 9 (*Acad9*) and cytochrome P450, family 2, subfamily u, polypeptide 1 (*Cyp2u1*) transcript expression were higher in *Ccdc93*^*+/-*^ and their functions are reported in fatty acid beta-oxidation and lipid metabolism, with increased activity associated with alteration of mitochondrial architecture, bioenergetics and oxidative stress [38–41].

**Fig 4:**
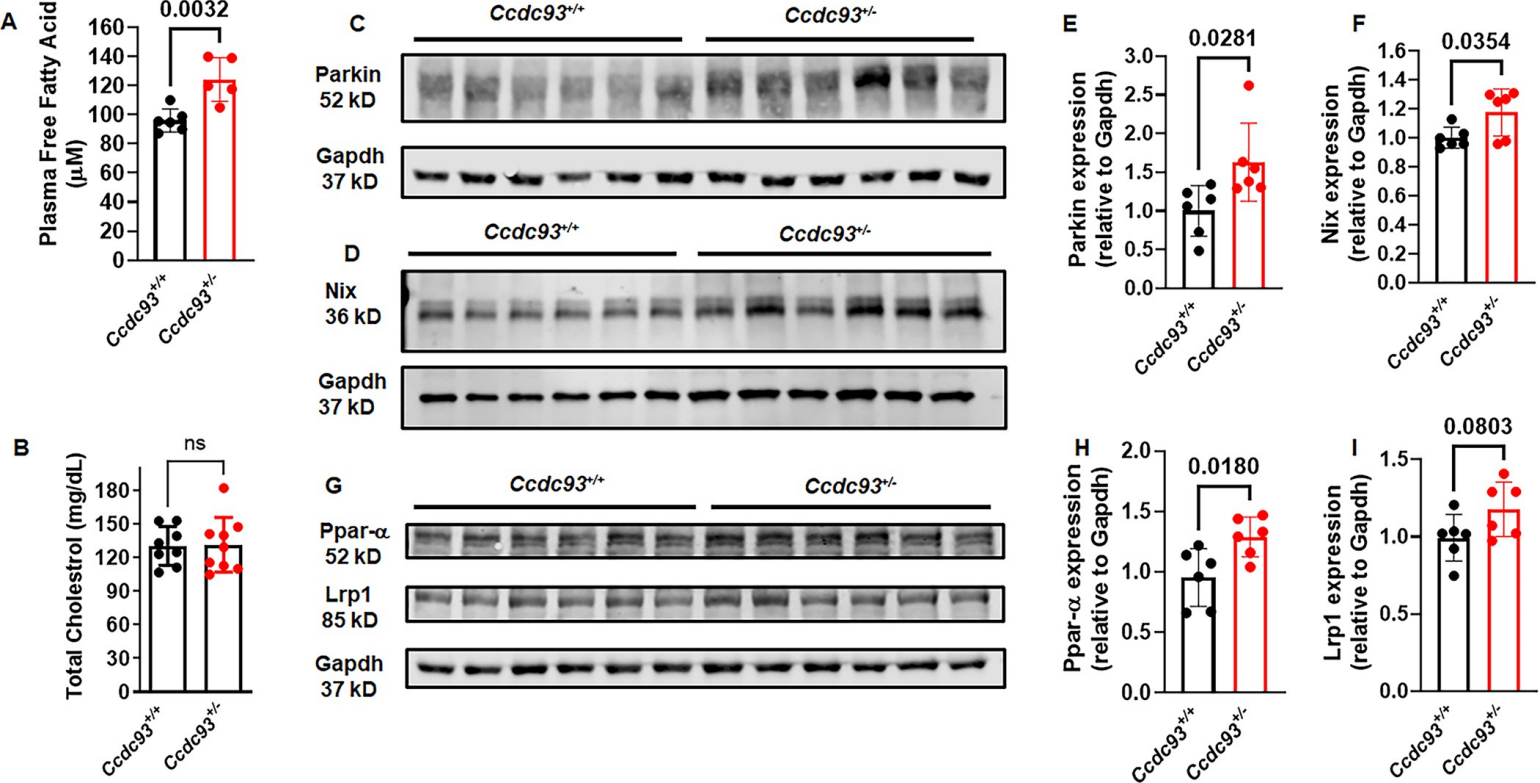
Plasma free fatty acid, aortic Parkin, Nix and Ppar-α protein expression were increased in *Ccdc93*^*+/-*^ mice. **(A)** Plasma free fatty acid was significantly increased in *Ccdc93*^*+/-*^ mice as compared to *Ccdc93*^*+/+*^ littermate controls. (**B**) Total plasma cholesterol was not different between the genotypes. **(C-F)** Aortic Parkin and Nix protein expression, markers of mitochondrial dysfunction, and **(G-H)** Aortic Ppar-α protein expression were significantly higher in *Ccdc93*^*+/-*^ mice as compared to *Ccdc93*^*+/+*^ littermate controls. **(G-I)** Aortic Lrp1 protein expression was higher in *Ccdc93*^*+/-*^ mice but did not reach statistical significance. *P*<0.05 was defined to be statistically significant. N = 5–6 in each group. Data are expressed as mean±s.d.

Based upon these results, we hypothesized that concordant energetic abnormalities would be observed in *Ccdc93*^*+/-*^ mice. Fat mass and weights of adipose tissues from three different anatomic locations (gonadal and inguinal white adipose tissue, and interscapular brown adipose tissue) were not statistically different between *Ccdc93*^*+/-*^ mice compared to *Ccdc93*^*+/+*^ littermate controls (**S12 Fig**) Circulating plasma free fatty acids (FFA) levels but not total cholesterol was significantly higher in *Ccdc93*^+/-^ mice as compared to littermate controls (**Fig 4A and 4B**). Concordant with the mouse data, human *CCDC93* rs33975708 A carriers displayed higher FFA levels (1.67-fold increase, *P* = 0.35, **S4 Table**) that was not statistically significant in this underpowered analysis, owing to the rarity of this genetic variant.

Liver weight relative to body weight was examined since the liver contributes to FFA pools in tissues and plasma [42], and this was mildly increased in *Ccdc93*^*+/-*^mice (**S12B Fig**). Analysis of liver histology by H&E showed no histologically evident lesions to explain the increased liver weight in *Ccdc93*^*+/-*^ mice (**S13 Fig**). Plasma glucose was similar in *Ccdc93*^*+/-*^ heterozygous mice as compared to the littermate *Ccdc93*^*+/+*^ control mice (**S12C Fig)**, consistent with our observation where *CCDC93* rs33975708 A carriers showed similar blood glucose levels as compared to non-carriers (**S4 Table**). Aortic Parkin and Nix protein, both of which are markers of mitochondrial dysfunction were significantly elevated in *Ccdc93*^*+/-*^ mice (**Fig 4C–4F**). Aortic peroxisome proliferator-activated receptor-alpha (Ppar-α) was significantly higher in *Ccdc93*^*+/-*^ mice as compared to littermate controls (1.28-fold higher, *P* = 0.018, **Fig 4G and 4H**), and low-density lipoprotein receptor-related protein (Lrp1) protein expression was higher but did not reach statistical significance (1.17-fold higher, *P* = 0.083, **Fig 4G and 4I**).

## Discussion

In an association study of exome-wide variants and BP traits in 5,954 Han Chinese individuals, we identified 4 trait-locus associations with 4 BP traits, including traditionally measured BP traits and cSBP, with cSBP suggested to be a more accurate predictor of cardiovascular diseases. We replicated previously reported associations with BP traits, and identified 2 novel associations, including the association of cSBP with rs33975708, a nonsynonymous variant in *CCDC93* (c.535C>T, p.Arg179Cys). This variant demonstrated pleiotropic associations with peripheral BP traits, Ba-PWV and LDL cholesterol, consistent with a recently described association of *CCDC93* genetic variation with cellular LDL receptor trafficking [43]. Replication studies of specialized measurements, in this case cSBP, and rare genetic variation are challenged by sample size requirements of the replication resources. Although we were able to leverage a few resources to define robust human genetic evidence supporting the association of rs33975708 and cSBP, we undertook studies of a genetic mouse model of *CCDC93* loss, as the variant in humans was predicted to lead to genetic deficiency of this gene. In functional validation experiments, mice with heterozygous loss of *Ccdc93* indeed demonstrated higher SBP than littermate control mice. In contrast, *Ccdc93*^*-/-*^ in its homozygous form was embryonic lethal, supporting its role in the embryonic development. *Ccdc93* expression was enriched in the vascular endothelium, and *Ccdc93*^*+/-*^ mice demonstrated endothelial dysfunction, evidenced by impaired acetylcholine mediated relaxation, as well as enhanced arterial contractility. Unbiased transcriptomic analysis demonstrated mitochondrial dysfunction, an effect that was validated in the elevated expression of Parkin and Nix, markers of mitochondrial dysfunction.

cSBP is associated with future cardiovascular events, and associations independent of pSBP have been defined [19,23,24,44]. In comparisons of the relationship of cSBP and pSBP measures with cardiovascular events, cSBP was superior to pSBP for the prediction of future cardiovascular events [45]. In addition to the CAFÉ study which reported differential effects on central pressure for different BP-lowering drugs despite similar effects on brachial pressure [17], a meta-analysis showed antihypertensive agents reduced pSBP more than cSBP (weighted mean difference [WMD] 2.52 mmHg, 95% CI 1.35 to 3.69) and β-Blockers posed a significantly greater reduction in pSBP as compared to cSBP (WMD 5.19 mmHg, 95% CI 3.21 to 7.18) [26]. cSBP measurement requires specialized techniques and has thus not been widely studied; we have thus studied cSBP in addition to traditional peripheral BP traits.

The coiled-coil domain containing 93 (*CCDC93*) gene encodes for a protein that is part of the “CCC” protein complex comprised of COMMD, CCDC22, and CCDC93. The recycling to the cell surface of Notch is dependent on the CCC complex [46,47]. Absence of *COMMD9* resulted in reduced Notch-dependent signaling, and deletion of *Commd9* led to embryonic lethality in mice due to abnormal cardiovascular development [47]. Similar to *Commd9* deletion [47], embryonic lethality of *Ccdc93* homozygous mice in our study supports an essential role in development at an early stage [47]. Both *CCDC93* and the CCC complex mediate endosomal sorting of the low-density lipoprotein receptor (*LDLR*) [43,48,49]. Transcriptome analysis of *Ccdc93*^*+/-*^ mice in our study notably identified alterations of adipogenesis and fatty acid metabolism, possibly linking the role of *Ccdc93* in endosomal trafficking of *Ldlr* and the low-density lipoprotein receptor-related protein (*Lrp1)*. In a previous genetic study of individuals of European ancestry, *CCDC93* (rs17512204; C>T, p.Pro228Leu) was associated with lower LDL-C levels, lower cardiovascular mortality, and lower risk of myocardial infarction [43]. Additional *CCDC93* variants have been associated with venous thromboembolism, triglyceride levels, and total cholesterol levels [50–52]. In the current study, another coding variant in *CCDC93* (rs33975708; c.535C>T, p.Arg179Cys), was associated with higher LDL-C and supports potential regulatory function of *CCDC93* in CVDs.

The cSBP-associated variant, rs33975708, has an overall MAF in the 1000 genomes reference of 4.07% but varies between populations, with the lowest in East Asians (0.00%) and the highest in Europeans (8.55%). Similarly, in the Exome Aggregation Consortium (ExAC) database, the frequency of this variant was 0.2% in individuals of East Asian ancestry and 7.43% in European-ancestry individuals.

In *Ccdc93*^*+/-*^ heterozygous mice, arterial *Ccdc93* transcript expression was reduced approximately 50% as expected, while protein expression was reduced by 22%, indicating that Ccdc93 protein expression is somewhat stabilized in these mice. Importantly for the objective of the current study, SBP elevation was observed in *Ccdc93*^*+/-*^ mice as compared to their *Ccdc93*^*+/+*^ littermate controls, confirming the direction of the discovered human genetic effect. Concordant endothelial-dependent and endothelial-independent relaxation impairment in *Ccdc93*^*+/-*^ heterozygous mice, along with increased contractile responses to vasoconstrictive challenges, suggested both endothelial and vascular smooth muscle defects in arterial function that result from *Ccdc93* deficiency. Transcriptome analysis defined mitochondrial dysfunction and expression of key reactive oxygen species (ROS) transcripts including *Mdh2*, *Xdh*, *Cyc1*, *Aldh5a1*, *Aldh1a7* increased in *Ccdc93*^*+/-*^ mice suggest mitochondrial-derived ROS production may mediate the observed hypertensive effects, as ROS has been implicated in the mechanisms of hypertension in other settings [53,54]. *Mdh2* is the final enzyme in the mitochondrial tricarboxylic acid cycle and its activity is associated with increased oxidative stress in the brain of Alzheimer disease [55]. *Xdh* is a source of superoxide ion, hydrogen peroxide, and nitric oxide, which can function as second messengers in various downstream vascular pathways [56]. *Xdh*-derived ROS are proinflammatory in the vasculature as they increase vascular permeability and arteriolar tone [57] and thus promote hypertension, cardiovascular diseases, and atherosclerosis [58,59].

Peroxisome proliferator-activated receptors-α (*Ppar-α*) is a member of the superfamily of ligand-activated nuclear transcription factors and its expression was significantly higher in *Ccdc93*^*+/-*^ mice, both at transcript- and protein-levels. *Ppar-α* has been implicated in the regulation of genes involved in mitochondrial fatty acid β-oxidation. The promoters of medium-chain acyl-CoA dehydrogenase and mitochondrial HMG-CoA synthase are *Ppar-α* responsive [60]. PPAR agonists are also involved in the production of reactive oxygen species [61]. Concordant with the transcriptome data of the current study of abnormal mitochondrial fatty acid metabolism, plasma FFA levels were significantly higher in *Ccdc93*^*+/-*^ mice, and a higher liver weight may suggest contribution to free fatty acid pools in tissues and plasma [42]. A positive correlation of mouse FFA levels was observed in our human samples. Human correlates of this finding include that elevated plasma FFA levels have been positively associated with SBP in nondiabetic subjects in a cross-sectional study [62]. Experimental FFA elevation achieved by intralipid infusion in non-obese women resulted in impaired capillary recruitment and acetylcholine-induced vasodilation in the skin microvasculature, as well as increased SBP [63]. Accumulating evidence suggests that FFAs contribute to hypertension by altering several pathways including alpha-adrenergic stimulation, increase in oxidative stress, stimulation of vascular cell growth, and endothelial dysfunction [64].

Mitochondrial dysfunction was validated through the examination of protein marker expression in *Ccdc93*^*+/-*^ mice as compared to controls. The protein Parkin is a E3-ubiquitous ligase that accumulates on the outer membrane of damaged mitochondria which ubiquitinates mitochondrial outer membrane proteins [65,66], and activates mitochondria for autophagy and degradation in lysosomes [67]. Nix is a mitochondrial protein and a member of the Bcl-2 family of apoptotic regulators and is implicated in the pathogenesis of cancer and heart disease by regulating mitophagy and cell death [68]. Nix also plays a critical role in Parkin-mediated mitochondrial autophagy by controlling the mitochondrial translocation of Parkin [69]. Evidence suggests that alterations in mitochondrial function and ROS production have been associated with endothelial dysfunction, development of hypertension, and cardiac hypertrophy [70]. The current study’s findings suggest that fatty acid metabolism and mitochondrial dysfunction play key roles in regulating BP and vascular function in *Ccdc93*^*+/-*^ mice.

The limitations of this study include a modest sample size, particularly compared to larger GWAS that have been carried out for peripheral BP traits. By using a custom exome chip array including Asian-specific nonsynonymous SNPs from sequenced samples in East Asian individuals, we identified a novel exonic variant association with cSBP that ideally would be replicated in large, independent samples. Due to the limited availability of ancestry-matched cohorts with cSBP measurement and the rarity of the discovered variant, replication experiments were under-powered, although using GRS, the complex genetic architecture of BP regulation in our cohort was aligned with that of previously reported BP GWAS. In order to further validate the effect of the variant identified, we undertook the generation and characterization of a novel transgenic mouse model deficiency in *Ccdc93*, which identified a similar phenotype and prioritized vascular mechanisms of BP regulation by *Ccdc93* for further study.

## Methods

### Ethics statement

All animal procedures were approved by University of Michigan Institutional Animal Care and Use Committee (IACUC), and mice were housed and cared for in accordance with the Guide for the Care and Use of Laboratory Animals.

### Discovery samples

In this study, subjects were recruited into the Peking University-University of Michigan Study of Atherosclerosis (PUUMA) in China, which is a large-scale project designed to study cardiovascular traits in China [22]. In total, 5,959 study participants of Chinese ancestry were genotyped in our BP study. Study protocols were approved by the Institutional Review Board (IRB) of Peking University First Hospital (IRB2014[816]) and Peking University Health Science center (IRB00001052-11086), and the study was approved by the University of Michigan IRB (HUM00074756). Written informed consent was collected from all subjects. Fasting venous blood samples were gathered for DNA genotyping and biochemical analyses. Medical and drug histories were accessed by standard questionnaire for each subject. Seated peripheral BP was measured for each participant with the standard method of calibration and appropriately sized cuffs after a 5-minute rest using an Omron HEM-7117 electronic sphygmomanometer. Triplicate measurements for each participant were taken with at least 1 minute between successive readings [22]. Noninvasive cSBP measurements were performed in a seated position using radial artery tonometry with an Omron HEM-9000AI device, as we have described and validated previously [22]. Brachial-ankle pulse wave velocity (Ba-PWV) was measured in the supine position after resting for at least 5 min (Omron Colin BP-203RPEIII; Omron Healthcare) by trained staffs. Total cholesterol (TC), triglycerides (TG), high-density lipoprotein cholesterol (HDL-C), low-density lipoprotein (LDL-C) and fasting blood glucose (FBG) were measured using Roche (Basel, Switzerland) C8000 Automatic Analyzer.

### Replication and validation study samples

Replication analyses of the discovered associations were carried out using multiple replication resources. For pSBP, we accessed customized Illumina ExomeChip genotyped SNPs from patients in a study of myocardial infarction [71] at Peking University First Hospital (N = 565) and Peking University Third Hospital (N = 127) in China. For cSBP, we evaluated associations in all available data from our discovery cohort resource. cSBP measurement was repeated 2.3 years after the first visit (N = 2,897 among 3,448 were included in the discovery stage), labeled “visit 2” cSBP. The baseline cSBP and visit 2 cSBP measurements were then averaged to generate a long-term averaged (LTA) [33] cSBP trait (N = 4,938). The additional LTA and Visit 2 data were analyzed for association with cSBP. In an independent cohort from Ji County in Tianjin, China, available subjects harboring the cSBP candidate variant were selected for cSBP measurement (N = 9), and their cSBP values were compared with age- and sex-matched individuals without the variant (N = 67 invited to participate in the study). In total, there were nine subjects with heterozygous *CCDC93* rs33975708-A allele. We used Student’s t-test and linear regression adjusting for age and sex to assess the differences of cSBP values between these two groups, and association of rs33975708-A with cSBP.

### Genotyping

Genotyping was conducted using a custom Illumina ExomeChip array with additional variants included in the design of the genotyping array based upon sequencing data from East Asian individuals, as has been described and used in other EWAS studies in China [71]. This exome array was designed including custom content representing 58,317 variants detected through genome sequencing of Asian individuals, in addition to the standard Infinium Human Exome Bead Chip (Illumina, CA) [72], which accessed a total of 302,218 variants. Genotype calling was performed using GenTrain version 2.0 in GenomeStudio V2011.1 (Illumina) independently for both cohorts, followed by cohort-specific quality control (QC).

### Data and sample quality controls

Sample and variant QC was performed as previously described [71], for evidence of bias or low-quality genotypes. Variants with low cluster score, Hardy-Weinberg equilibrium deviations *P* < 0.0001 and call rate < 99% were excluded. Variants with a minor allele count larger than 3 were included. For the sample QC, we excluded duplicates based on whole genome genotyping data IBD (Identical by descent) analysis, gender miss-matched samples, and samples with missing phenotypes. All samples had missing call rate < 1%. None of them failed QC in inbreeding coefficient check. Finally, 5,954 samples (2,185 males, 3,768 females) and 86,148 SNPs were retained after the QC in this BP EWAS. The correlation r^2^ of the allele frequencies between PUUMA Chinese samples and 1000 Genomes East Asian samples was 0.98.

### Phenotypes and transformation

Our study phenotypes focused on BP measurements: pSBP, pDBP, pMAP and cSBP. Three repetitive measurements were recorded for pSBP (ex. SBP1, SBP2, SBP3) and pDBP (ex. DBP1, DBP2, DBP3), and the average number was used in the further analysis. Approximately 33% of individuals in the PUUMA cohort were taking anti-hypertensive medications, and their pSBP and pDBP measurements were adjusted +15 mm Hg and +10 mm Hg, respectively, before further analysis. pMAP was from (1/3 × pSBP) + (2/3 × pDBP). Distributions of pSBP, pDBP, pMAP, and cSBP were inspected, and outliers were detected as >3 standard deviations (s.d.) from the mean for each trait. For pSBP, pDBP, pMAP, and cSBP, the phenotypic values were substituted by the residual values using technique of linear regression adjustments: Trait ~ age + age^2^+ body mass index (BMI) sex.

### Generation of *Ccdc93* heterozygous mice

The loss of *CCDC93* was modeled in a new transgenic mouse after CRIPSR/Cas9 targeting leading to an out-of-frame deletion in the *Ccdc93* gene. *Ccdc93* heterozygous mice (*Ccdc93*^+/-^) carrying a 21-nucleotide deletion were generated by injecting Cas9 mRNA, a guide RNA, and a donor DNA (Biomatrik, Ontario, Canada) into the embryo obtained by mating (C57BL/6 X SJL) F1 or C57BL/6 female mice with (C57BL/6 X SJL) F1 male mice. Pronuclear microinjection was performed as previously described [73]. *Ccdc93*^+/-^ mice were mated with C57BL/6 mice and 129/Sv mice (Taconic Biosciences, NY, USA) for at least 10 generation to confirm germline transmission. Genotyping of *Ccdc93*^+/-^ mice was performed by PCR amplifying the region using forward (5’GGAAGGGTGGGAGCGAGGAG3’) and reverse primer (5’ GTTGGCTTGCTTGCTTCTTTCTTTC 3’) and confirmed by sanger sequencing (Genewiz, NJ), **S5 Fig.**

### BP measurement in mice

Male and female *Ccdc93*^*+/*-^ (129/Sv) mice and their littermate controls at 12–16 weeks of age underwent SBP measurement by non-invasive tail-cuff plethysmography (BP-2000, Visitech Systems, NC) as previously described [74]. BP was measured after keeping trained mice quietly on a temperature-controlled restrainer for 10 minutes. Then, ten preliminary cycles were performed to allow the mice to acclimate and warm up to increase the blood-flow to the tail for optimal BP signals followed by 10 more cycles for the actual measurement. BP measurements were performed between 10 am and 12 noon. Mice received regular chow and water ad libitum.

### Endothelial cell-specific translating ribosome affinity purification (EC-TRAP)

EC-TRAP was performed as we have previously described [75]. Briefly, *Rpl22*^*fl/fl*^, *Tek*^*+/0*^ C57BL/6 wild-type mice were perfused with cycloheximide (100 μg/ml, Sigma-Aldrich) to stop protein translation and stabilize ribosomal complexes, and tissues were snap frozen until further use. Frozen tissues were homogenized using a Dounce homogenizer in lysis buffer [75] (50 mM Tris pH 7.4, 100 mM KCl, 12 mM MgCl2, 1% Igepal CA-630, Protease inhibitor, 200 U/ml RNAse OUT, 1 mg/ml heparin, 1 mM dithiothreitol and 100 μg/mL cycloheximide). Cellular debris was removed by centrifugation and clear lysates were incubated with a purified mouse monoclonal antibody against the HA epitope tag (HA.11 clone 16B12, BioLegend, CA; 3μg/400μl lysate) for 1 hour at 4°C. Protein G magnetic beads (New England BioLabs, MA; 100μl/400μl lysate) equilibrated in polysome buffer were then added for an additional 30-minute incubation. The magnetic beads were subsequently washed 3 times with high salt buffer (50 mM Tris pH 7.4, 300 mM KCl, 12 mM MgCl2, 1% Igepal CA-630, 1 mM dithiothreitol and 100 μg/mL cycloheximide). RA1 buffer (NucleoSpin RNA, Takara Bio USA, CA) supplemented with 2-mercapto-ethanol (1% vol/vol) was added to the beads and vigorously vortexed to dissociate the mRNA from the HA-tagged polysomes. Total RNA from tissue lysate and TRAP polysomal complexes were isolated using NuceloSpin RNA kit (Takara Bio USA, CA), including an on-column DNase digestion step and RNA was quantified by Quant-it RiboGreen RNA assay (Thermo Fisher Scientific, MA). *Ccdc93* transcript expression was queried in the mouse single-cell RNA-seq database at Tabula Muris with *Tek* used to identify aortic endothelial cells (https://tabula-muris.ds.czbiohub.org/) and human single-cell RNA-seq GTEx database at Protein Atlas (https://www.proteinatlas.org/).

### RNA-Seq library preparation, sequencing, and qRT-PCR

To assess aortic transcriptional changes in *Ccdc93*^+/-^ transgenic mice, RNA-Seq was performed on descending thoracic aortae from male and female *Ccdc93*^+/-^ heterozygous mice (N = 3 per sex) and their *Ccdc93*^+/+^ littermate controls (N = 3 per sex). Total RNA was isolated using Trizol and chloroform extraction, followed by RNA purification as described above. RNA samples meeting RIN score >7 was included in the RNA-Seq. All libraries were prepared with 10 ng of RNA using SMART-Seq V4 kit for low RNA input (Takara Bio USA, CA). After quality control, all samples were pair-end sequenced by NovaSeq 6000 in MedGenome (Foster City, CA), with a read length of 100 bp and 40 million read depth per sample. Aortic *Ccdc93* RNA expression in *Ccdc93*^+/-^ heterozygous and *Ccdc93*^+/+^ littermate controls mice was measured as described previously [76]. Briefly, total aortic RNA was extracted and reverse transcription of 200 ng of total RNA was performed using SuperScript-III first-strand synthesis system (ThermoFisher, MA, USA) in a total volume of 20 μL. The exon spanning primers of *Ccdc93* and *Gapdh* were designed (primer 3 software, **S5 Table**) and synthesized (Invitrogen, MA). cDNA was amplified using PCR master mix with SYBR-Green (Applied Biosystems, MA) and data were calculated by 2^- ΔΔCT^ method [77] and presented as fold change of transcripts of *Ccdc93* in mouse aortae and normalized over housekeeping *Gapdh* gene, as compared to control samples.

### RNA-Seq analysis of differential gene expression

All data were aligned to the reference genome GRCh37 (hg19) by STAR aligner (2.7.0) with the annotation from GENCODE (GRCm39) [78,79]. Raw counts were extracted and then normalized with DESeq2 [80]. After quality assessment, analyses were performed in DESeq2 to determine differential transcript expression between *Ccdc93*^+/-^ and *Ccdc93*^+/+^ littermate control groups. Gene ontology enrichment analysis and Gene set enrichment analysis were performed using DAVID Bioinformatic Resources, and by Hallmark pathways set files from the molecular signatures database, respectively [81,82]. The significant threshold was defined as false discovery rate (FDR) < 0.1.

### Histological examination of fetal placental units, adult liver, and arterial section

Uterine horns of *Ccdc93*^+/-^ X *Ccdc93*^+/-^ mice subjected to timed mating were stored at E10.5 in 4% neutral buffered formalin until histologic analysis. The uterus was not opened but was cut into sections with 2 fetoplacental units (FPU) or resorption sites per section. Each section of two FPU or resorption sites were processed on an automated histology processor (Tissue Tek VIP, Sakura, Torrance, CA). Sections were embedded in paraffin with sections oriented laterally to enable visualization of both mesometrial and antimesometrial sides of the tissue. Liver tissues of adult *Ccdc93*^*+/-*^ and littermate controls were paraffin embedded. Aorta and mesenteric artery of wild-type 129/Sv mice were paraffin embedded for immunohistochemical analysis by Ccdc93 antibody (1:200 dilution, 20861-1-AP, Proteintech) and endothelial specific markers platelet and endothelial cell adhesion molecule-1 (PECAM-1, 1:50 dilution, DIA-310, Dianova, Switzerland). Detection of Ccdc93 and PECAM-1 protein expression is based on horseradish peroxidase (HRP) catalysis of a 3,3′-Diaminobenzidine (DAB, brown) chromogenic reaction, with a hematoxylin (blue) nuclear counterstain. Sections of adult liver, artery and FPUs were cut from each block at 4 μm thickness. Pixel quantification of Ccdc93 protein expression was performed using ImageJ (NIH) and pixel intensity was normalized by arterial area encompassing endothelial cells and non-endothelial cells. Slides were evaluated by a board-certified veterinary pathologist using a BX45 Olympus light microscope. Slides were digitized to pyramidal Tiff files on a digital slide scanner (Leica Aperio AT2, Leica Biosystems) and representative images were taken from digitized slide files using freely available manufacturer-provided software (Leica Aperio ImageScope). Composite images were assembled in Adobe Photoshop CC (v 19.0, Adobe Systems Inc). Photo processing was confined to global adjustment of image size, white balance, brightness, or contrast that did not materially alter the interpretation of the image.

### Vascular reactivity analysis using wire myography

Vascular reactivity assessed by wire myography was performed as previously described [83]. Briefly, descending thoracic aorta of *Ccdc93*^+/-^ and *Ccdc93*^+/+^ mice were carefully dissected to remove perivascular fat and cut into 2 mm rings. Rings were transferred into physiological saline solution (PSS, 37°C gassed with 95% air and 5% CO_2,_ PSS solution: 130 mM NaCl, 4.7 mM KCl, 1.17 mM MgSO_4_.7H_2_O, 14.9 mM NaHCO_3_, 1.18 mM KH_2_PO_4_, 0.026 mM EDTA, 1.6 mM CaCl_2_, and 5 mM glucose, pH 7.4) and mounted on pins in multi-wire myograph connected to a force transducer (DMT 620M, MI) to measure wall tension developed by the rings. Prior to assessing vascular function, aortic rings were pre-treated with high KCl buffer (60 mM) to induce contraction and to determine maximal contractility, and this was used for normalizing force generated by dose response of phenylephrine. Vascular function was evaluated by contraction induced by phenylephrine (10 μM) and vasodilation induced by acetylcholine (Ach, 10 μM) in aorta pre-contracted with phenylephrine (10 μM). Dose responses of acetylcholine and sodium-nitroprusside (1 nM to 10 μM) of aorta pre-contracted by phenylephrine were then recorded and analyzed. Rings were washed three times with physiological saline solution and equilibrated for 30 minutes at 37°C between each successive vasoactive treatment.

### Plasma free fatty acid, glucose measurement, and body composition

Blood was collected from *Ccdc93*^+/-^ and *Ccdc93*^+/+^ mice from the abdominal vena cava into citrate containing tubes (0.4% w/v) and centrifuged at 2,500 rpm for 15 minutes at 4°C to obtain plasma which was stored at -80°C until further use. Plasma free fatty acid level (FFA) in mouse and human samples was measured using commercially available sensitive enzyme-based assay (Abcam 65341) in which fatty acids are converted to their CoA derivatives in the presence of added acyl-CoA synthetase. Acyl-CoA products were then subsequently oxidized leading to the formation of color which was measured colorimetrically at 570 nm. Total plasma cholesterol in mice was measured by colorimetric detection at 450 nm by enzyme-based assay (Abcam 285242).

Fasting blood glucose level in human samples was measured and analyzed at the central laboratory of Tianjin Medical University General Hospital as previously described [34]. Mouse plasma glucose was measured using commercially available enzyme-based assay (K039-H1, Arbor Assays, MI). Glucose oxidase reacts with glucose to produce hydrogen peroxide, which, in the presence of HRP, reacts with the substrate to generate a colored product which was measured at 560 nm. Body composition scans in *Ccdc93*^*+/-*^ and littermate controls were performed at the University of Michigan Mouse Metabolic and Phenotypic Center (MMPC). Body fat and lean mass were measured using a nuclear magnetic resonance (NMR)-based analyzer (EchoMRI, 4in1-500, Houston, TX). The measurements were performed in under 2 minutes while conscious mice were placed individually in the measuring tube.

### Western blot

Aortic tissue protein (20 μg) from *Ccdc93*^+/-^ and *Ccdc93*^+/+^ mice were separated by sodium dodecyl sulfate-polyacrylamide gel (12% SDS-PAGE) and transferred on to a nitrocellulose membrane. The blotted membranes were incubated with rabbit anti-Ccdc93 antibody (1:1000, 20861-1-AP, Proteintech), mouse anti-parkin (1:2000, ab77924, Abcam), rabbit anti-Nix/BNIP3L (1:1000, #12396S, CST), rabbit anti-Ppar-α (1:1000, ab126285, Abcam) and rabbit anti-Lrp1 (1:1000, # 64099, CST). The housekeeping gene product Gapdh (1:10,000, # 97166, CST) served as a loading control. Protein bands were visualized using a fluorescent-conjugated secondary antibody (anti-rabbit IRDye 680RD, anti-mouse IRDye 800CW, LI-COR Biosciences, Lincoln, NE) and imaged using LI-COR Odyssey CLx imaging system. Densitometric analysis of protein bands were quantified using NIH ImageJ software.

### Statistical analyses

A mixed linear model considering kinship was performed using the EMMAX program [84], which is a statistical test for large scale human or model organism association mapping accounting for the sample structure. As described above, we adjusted for age, age^2^, BMI, and sex for our phenotypes first and run mixed linear model based on the residuals and the exome-wide SNPs. 5,954 samples and 86,148 SNPs were tested in the discovery stage and two novel identified SNPs with *P* <5.8 × 10^−7^ (0.05/86,148) were re-examined in replication stage. Manhattan plots were generated from exome-wide SNPs, with marked of known BP GWAS ± 500 Kb region as positive controls and to filter the newly discovered loci. Quantile-quantile plots were depicted for SNPs in different minor allele frequency groups. λ_GC_ was evaluated for evidence of population stratification.

A published BP GWAS finding that included more than one million people was used to construct BP weighted genetic risk score (GRS), where SNPs were weighted according to BP coefficients from the GWAS discovery results(5). Genome-wide summary statistics of SBP and DBP were downloaded from the GWAS Catalog. Variants that achieved a *P*-value less than 5×10^−8^ from GWAS of the same trait were selected and LD-pruned (R^2^>0.2 in a 500Kb window). Their beta estimates were then aligned with our study’s summary statistic based on the identical effect alleles. The GTX package (https://www.rdocumentation.org/packages/gtx/versions/0.0.8) in R was applied to test the association between PRS of known BP and our BP analysis using GWAS summary statistics adjusted for age, age^2^, sex, BMI, and principal components (PCs).

Associations of novel BP-related SNPs with risk factors of coronary artery disease (including blood lipids, BP traits, pulse wave velocity, and body mass index) were tested using multivariate linear regression, with models adjusting for sex and age, using R program. For the analyses of blood lipids, those who were taking lipid-lowering drugs were excluded.

For experiments involving mice, all data were reported as mean±s.d. Student’s t tests (unpaired, two-tailed) were used to compare the significance between the two groups. *P*<0.05 was defined to be statistically significant.

## Supporting information

S1 Table GRS_BP_ association in PUUMA cohort. pSBP, peripheral systolic blood pressure; pDBP, peripheral diastolic blood pressure; pMAP, peripheral mean arterial pressure; cSBP, central systolic blood pressure.
(XLSX)

S2 Table Validation analyses of rs2165468-pSBP and rs33975708-cSBP associations. (XLSX)

S3 Table Complete blood counts in *Ccdc93*^*+/-*^ and *Ccdc93*^*+/+*^ mice. WBC, white blood cell; NE, neutrophils; LY, lymphocyte; MO, monocyte; EO, eosinophil; BA, basophil; RBC, red blood cell; Hb, hemoglobin; HCT, hematocrit; MCV, mean corpuscular volume; MCH, mean corpuscular hemoglobin; MCHC, mean corpuscular hemoglobin concentration; RDW, red cell distribution width; PLT, platelet; MPV, mean platelet volume; K, thousand; M, million; g, gram; μL, microliter, dL, deciliter; fL, femtoliter.
(XLSX)

S4 Table Human plasma free fatty acid and blood glucose levels. In human, *CCDC93* rs33975708 A carriers displayed higher FFA levels as compared to non-carriers. Fasting blood glucose level was similar between *CCDC93* rs33975708 A carriers and non-carriers.
(XLSX)

S5 Table qRT-PCR primers. (XLSX)

S1 Fig Exome-wide association study (EWAS) for peripheral systolic blood pressure (pSBP). ExomeChip SNPs meeting quality control were analyzed in the EWAS. Results are shown in a **(A)** Manhattan plot and **(B)** Quantile-quantile (QQ) plot; the λ_GC_ value was 1.0. **(C)** Regional association plots with gene annotation for the chromosomes 10p15.2 region associated with pSBP is shown with the index SNP rs2165468 and additional SNPs within 500 kbp in each direction. Top panel: Regional pSBP association plot for the *PITRM1-KLF6* locus. LD (linkage disequilibrium) was calculated from our samples. Non-synonymous variants were annotated using ANNOVAR. The genetic recombination rate is based on Hapmap release 22. Middle Panel: Standardized varLD scores illustrate LD variations between populations (CEU vs. JPT+CHB, CEU vs. YRI, YRI vs. JPT+CHB) using genome positions from the Hapmap 3 reference. The red line represents the comparison between CEU (European ancestry) and JPT+CHB (East Asian ancestry), the purple line indicates CEU versus YRI (African ancestry), and the green line represents YRI versus JPT+CHB. Bottom Panel: Gene/transcript annotations are sourced from the reference downloaded from the UCSC database (EST, mRNA, uniGene, Encode, and RefGene).
(TIF)

S2 Fig EWAS for peripheral diastolic blood pressure (pDBP). ExomeChip SNPs meeting quality control were analyzed in the EWAS. Results are shown in a **(A)** Manhattan plot and **(B)** QQ plot; the λ_GC_ value was 1.0. **(C)** Regional association plots with gene annotation for the chromosomes 4q21.21 region associated with pDBP is shown with the index SNP rs13149993 and additional SNPs within 500 kbp in each direction. Top panel: Regional pDBP association plot for the *PRDM8-FGF5* locus. LD was calculated from our samples. Non-synonymous variants were annotated using ANNOVAR. The genetic recombination rate is based on Hapmap release 22. Middle Panel: Standardized varLD scores illustrate LD variations between populations (CEU vs. JPT+CHB, CEU vs. YRI, YRI vs. JPT+CHB) using genome positions from the Hapmap 3 reference. The red line represents the comparison between CEU (European ancestry) and JPT+CHB (East Asian ancestry), the purple line indicates CEU versus YRI (African ancestry), and the green line represents YRI versus JPT+CHB. Bottom Panel: Gene/transcript annotations are sourced from the reference downloaded from the UCSC database (EST, mRNA, uniGene, Encode, and RefGene).
(TIF)

S3 Fig EWAS for peripheral mean arterial pressure (pMAP). ExomeChip SNPs meeting quality control were analyzed in the EWAS. Results are shown in a **(A)** Manhattan plot and **(B)** QQ plot; the λ_GC_ value was 1.0. **(C)** Regional association plots with gene annotation for the chromosomes 4q21.21 region associated with pMAP is shown with the index SNP rs13149993 and additional SNPs within 500 kbp in each direction. Top panel: Regional pMAP association plot for the *PRDM8-FGF5* locus. LD was calculated from our samples. Non-synonymous variants were annotated using ANNOVAR. The genetic recombination rate is based on Hapmap release 22. Middle Panel: Standardized varLD scores illustrate LD variations between populations (CEU vs. JPT+CHB, CEU vs. YRI, YRI vs. JPT+CHB) using genome positions from the Hapmap 3 reference. The red line represents the comparison between CEU (European ancestry) and JPT+CHB (East Asian ancestry), the purple line indicates CEU versus YRI (African ancestry), and the green line represents YRI versus JPT+CHB. Bottom Panel: Gene/transcript annotations are sourced from the reference downloaded from the UCSC database (EST, mRNA, uniGene, Encode, and RefGene).
(TIF)

S4 Fig Blood pressure traits, brachial-ankle pulse wave velocity and low-density lipoprotein in individuals with and without the rs33975708 cSBP increasing allele. **(A)** Peripheral systolic blood pressure (pSBP); (**B)** Peripheral diastolic blood pressure (pDBP); **(C**) Peripheral pulse pressure (pPP); (**D**) Peripheral mean arterial pressure (pMAP); (**E**) Brachial-ankle pulse wave velocity (Ba-PWV); and (**F**) Low-density lipoprotein-cholesterol (LDL-C). All these traits were significantly higher in *CCDC93* risk allele A carriers as compared to non-carriers.
(TIF)

S5 Fig Generation and DNA chromatogram of transgenic *Ccdc93*^*+/-*^ mice. **(A)** *Ccdc93* heterozygous mice were generated using the CRISPR/Cas9 system. **(B)** *Ccdc93*^*+/-*^ carried a 21-nucleotide deletion. The molecular weight of DNA bands after PCR amplification of the *Ccdc93* gene was not distinguishable by agarose gel electrophoresis. **(C)** Sanger sequencing of genomic DNA of *Ccdc93* showed difference between littermate control and heterozygous of *Ccdc93* with expected peak-on-peak DNA chromatogram observed in *Ccdc93*^*+/-*^ at the transgenic site (yellow highlight).
(TIF)

S6 Fig Embryonic lethality of *Ccdc93* homozygous deletion. **(A)** Genotype frequencies at birth and at E10.5 of *Ccdc93*^*+/-*^ X *Ccdc93*^*+/-*^ mating. At birth, homozygous *Ccdc93*^*-/-*^ mice were not viable and time mating at E10.5 of embryos produced from *Ccdc93*^*+/-*^ X *Ccdc93*^*+/-*^ mating showed *Ccdc93* homozygosity were embryonic lethal and died before E10.5 of gestation. **(B)** Histology of fetoplacental units showed uterine horns from a *Ccdc93*^*+/-*^ (heterozygous) pregnant female mouse at day E10.5 of gestation. This pregnancy was the product of *Ccdc93*^*+/-*^ X *Ccdc93*^*+/-*^ timed mating. *Ccdc93*^*+/-*^ heterozygous embryos appeared viable (no evidence of necrosis, inflammation, or hemorrhage). **(C)** 2 resorption sites of *Ccdc93*^*-/-*^ homozygous contained maternal hemorrhage and necrotic debris accompanied by neutrophilic and lymphocytic inflammation within and around chorioallantoic membranes on the antimesenterial (fetal) side. There were no embryos or embryo tissues present.
(TIF)

S7 Fig Tissue weight of *Ccdc93*^*+/-*^ and *Ccdc93*^*+/+*^ mice. Tissue weight (**A**-heart weight, **B**-kidney weight, **C**-spleen weight) normalized over body weight were not different between the groups (N = 6–8 in each group). All data are shown as mean±s.d.
(TIF)

S8 Fig Aortic morphology and collagen content of *Ccdc93*^*+/-*^ mice. **(A-C)** Picrosirius red staining (PSR) for total collagen content of the descending thoracic aorta of littermate *Ccdc93*^*+/+*^ and **(D-F)** *Ccdc93*^*+/-*^ mice demonstrate no significant differences. Hematoxylin and eosin (H&E) staining of the descending thoracic aorta of **(G-I)** littermate *Ccdc93*^*+/+*^ and **(J-L)** *Ccdc93*^*+/-*^ mice did not demonstrate abnormal vascular morphology. Each section was examined under 4x objective microscope (Nikon’s Eclipse E600), photographed with a digital camera (DS-Ri1, Nikons Instrument), and evaluated by the ImageJ analysis system (NIH). PSR staining was used to facilitate automatic detection in pixel intensity by the image processing macro in ImageJ. N = 6 in each group. Scale bars 200 μm. All data are shown as mean±s.d.
(TIF)

S9 Fig Vascular cell-specific transcript expression by EC-targeted translating ribosomal affinity purification (EC-TRAP). **(A-B)** Endothelial cell (EC)-specific enrichment and vascular smooth muscle-specific (VSMC) depletion of transcript markers by EC-TRAP in C57BL/6 wild-type mice in **(A)** thoracic aortae and **(B)** mesenteric arteries *in vivo*. Total RNA from was isolated from input and anti-HA beads (IP-HA) from descending thoracic aortae and mesenteric arteries of *Rpl22*^*fl/fl*^, *Tie2-*^*Cre+/0*^ (Tie2-RiboTag mouse) and showed expected enrichment of EC-specific transcripts (*Tek*, *Cdh5 and Vwf*) and depletion of vascular smooth muscle-specific markers (*Cnn1*, *Acta2* and *Tagln*) in the IP-HA fraction as compared to input. *Ccdc93* transcript expression in the Tek subpopulations (EC-specific) was enriched (Fig 2J), confirming predominantly EC expression. N = 3–4 in each group. All data are shown as mean±s.d.
(TIF)

S10 Fig RNA-seq analysis of *Ccdc93*^*+/-*^ aortic transcriptomes. **(A)** Principal component analysis (PCA) plot showed no clear clustering by the genotype. The first and second principal components (PC1 and PC2) accounted for 61% and 19% variability in the RNA-Seq dataset. **(B)** Aortic *Ccdc93* transcript expression was significantly reduced in *Ccdc93*^*+/-*^ as compared to *Ccdc93*^*+/+*^ in bulk RNA-seq analysis, validating qPCR findings (**Fig 2C**). **(C)** Gene set enrichment analysis showed hallmark pathway associations with altered adipogenesis and fatty acid metabolism that were significantly upregulated in the thoracic aortae of *Ccdc93*^*+/-*^ mice as compared to *Ccdc93*^*+/+*^ littermate controls, false discovery rate (FDR<0.1).
(TIF)

S11 Fig SBP measurement in male and female *Ccdc93*^*+/-*^ and *Ccdc93*^*+/+*^ mice. A similar increase in SBP was observed in male and female *Ccdc93*^*+/-*^ heterozygous mice as compared to littermate controls. All data are shown as mean±s.d.
(TIF)

S12 Fig Body composition and plasma glucose level in *Ccdc93*^*+/-*^ and *Ccdc93*^*+/+*^ mice. **(A)** Body weight, and lean mass measured by EchoMRI were significantly higher in *Ccdc93*^*+/-*^ mice as compared to *Ccdc93*^*+/+*^ control mice. **(B)** A mild increase in the liver weight of *Ccdc93*^*+/-*^ heterozygous mice was observed, however adipose tissue weight (gonadal and inguinal white adipose tissue, and interscapular brown adipose tissue) were not different between the genotype groups. **(C)** Plasma glucose level was equivalent in *Ccdc93*^*+/-*^ heterozygous mice as compared to littermate control mice. Equal numbers of male and female mice (N = 4 per sex per genotype) were analyzed. White adipose tissue (WAT), brown adipose tissue (BAT). All data are shown as mean±s.d.
(TIF)

S13 Fig Representative liver histology of *Ccdc93*^*+/-*^ and *Ccdc93*^*+/+*^ mice. Hematoxylin and eosin stain (H&E) stained images are shown. No histological lesions were observed in the liver of *Ccdc93*^*+/-*^ mice as compared to the littermate controls. **(A)** Male *Ccdc93*^*+/+*^ wild-type control liver samples (inset-male wild-type biological replicates, N = 3). **(B)** Male *Ccdc93*^*+/-*^ heterozygous liver samples (inset-male *Ccdc93*^*+/-*^ heterozygous biological replicates, N = 3). **(C)** Female *Ccdc93*^*+/+*^ wild-type control liver samples (inset-female wild-type biological replicates, N = 3). **(D)** Female *Ccdc93*^*+/-*^ heterozygous liver samples (inset-female *Ccdc93*^*+/-*^ heterozygous biological replicates, N = 3). Scale bars 200 μm, insets 2 mm.
(TIF)

S14 Fig Evaluation of *CCDC93* expression by single-cell RNA-sequencing analysis. **(A-B)** *Ccdc93* expression was queried in the mouse scRNA-seq database Tabula Muris (https://tabula-muris.ds.czbiohub.org/), and in **(C)** human scRNA-seq GTEx database protein atlas (https://www.proteinatlas.org/), which showed low tissue specificity of *Ccdc93*, and validated *Ccdc93* expression in mouse aortic endothelial cells (blue dots in left figure panel A), shown in the tSNE plots, and enrichment of *CCDC93* expression in human liver vascular endothelial cells (colored dot in panel C).
(TIF)

S15 Fig Immunohistochemistry of Ccdc93 in mouse aorta. **(A-C)** Ccdc93 protein expression was observed in both endothelial and non-endothelial cells in wild-type mouse aortae (129/Sv). Three biological replicates of mouse thoracic aorta showed Ccdc93 expression (brown stained). **(D)** Pixel quantification showed 1.33-fold higher Ccdc93 protein expression in the endothelium (normalized by area) as compared to non-endothelial cells (*P* = NS). Scale bar = 200μm.
(TIF)

S16 Fig Immunohistochemistry of positive and negative controls of PECAM-1 (CD31) and Ccdc93 in mouse aorta. **(A)** Mouse aorta immunostaining of platelet and endothelial cell adhesion molecule-1 (PECAM-1), **(B)** Negative control (no antibody) immunostaining of mouse aorta serial section. **(C)** Positive control of mouse aorta immunostaining of Ccdc93. **(D)** Negative control of mouse aorta where Ccdc93 antibody was absent. Detection of PECAM-1 (black arrows) and Ccdc93 protein expression (black arrows represents EC expression and blue arrows represents medial expression) is based on horseradish peroxidase (HRP) catalysis of a 3,3′-Diaminobenzidine (DAB, brown) chromogenic reaction, with a hematoxylin (blue) nuclear counterstain, scale bar = 200μm.
(TIF)
