# Supplementary figures and images for: Genetic variation in CCDC93 is associated with elevated central systolic blood pressure, impaired arterial relaxation, and mitochondrial dysfunction
Source: PLoS Genet. 2024 Sep 9;20(9):e1011151. doi: 10.1371/journal.pgen.1011151 (PMC11421807; doi:10.1371/journal.pgen.1011151)

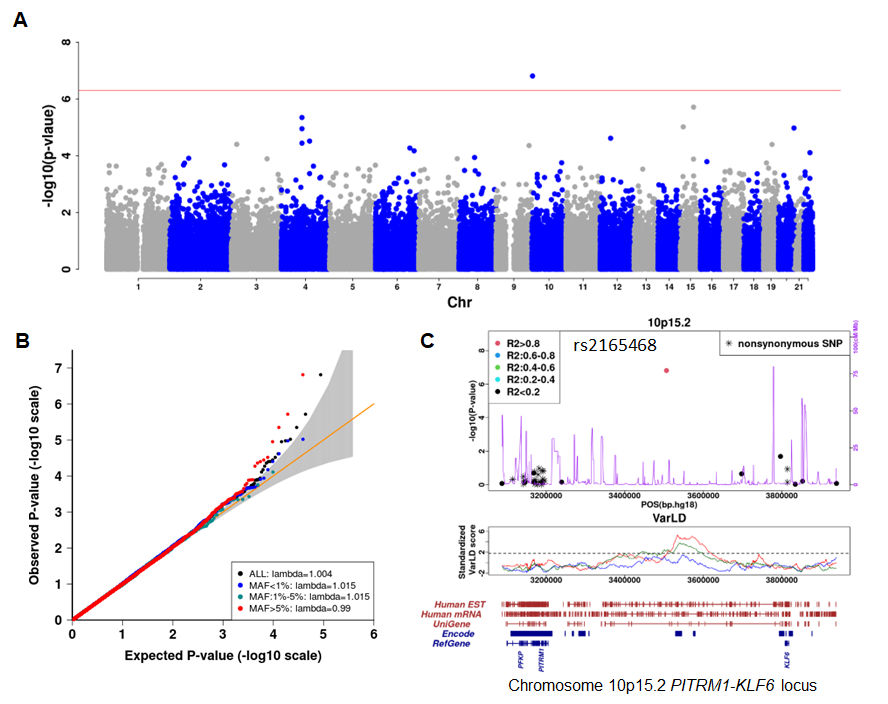

Supplement: S1 Fig — ExomeChip SNPs meeting quality control were analyzed in the EWAS. Results are shown in a (A) Manhattan plot and (B) Quantile-quantile (QQ) plot; the λGC value was 1.0. (C) Regional association plots with gene annotation for the chromosomes 10p15.2 region associated with pSBP is shown with the index SNP rs2165468 and additional SNPs within 500 kbp in each direction. Top panel: Regional pSBP association plot for the PITRM1-KLF6 locus. LD (linkage disequilibrium) was calculated from our samples. Non-synonymous variants were annotated using ANNOVAR. The genetic recombination rate is based on Hapmap release 22. Middle Panel: Standardized varLD scores illustrate LD variations between populations (CEU vs. JPT+CHB, CEU vs. YRI, YRI vs. JPT+CHB) using genome positions from the Hapmap 3 reference. The red line represents the comparison between CEU (European ancestry) and JPT+CHB (East Asian ancestry), the purple line indicates CEU versus YRI (African ancestry), and the green line represents YRI versus JPT+CHB. Bottom Panel: Gene/transcript annotations are sourced from the reference downloaded from the UCSC database (EST, mRNA, uniGene, Encode, and RefGene). (TIF) [file pgen.1011151.s006.tif]

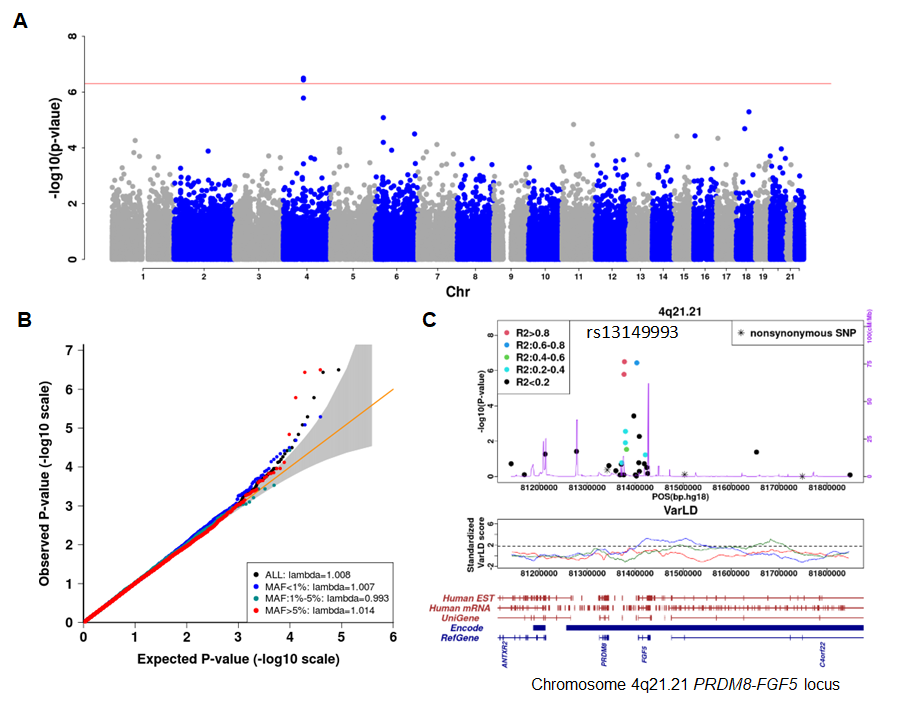

Supplement: S2 Fig — ExomeChip SNPs meeting quality control were analyzed in the EWAS. Results are shown in a (A) Manhattan plot and (B) QQ plot; the λGC value was 1.0. (C) Regional association plots with gene annotation for the chromosomes 4q21.21 region associated with pDBP is shown with the index SNP rs13149993 and additional SNPs within 500 kbp in each direction. Top panel: Regional pDBP association plot for the PRDM8-FGF5 locus. LD was calculated from our samples. Non-synonymous variants were annotated using ANNOVAR. The genetic recombination rate is based on Hapmap release 22. Middle Panel: Standardized varLD scores illustrate LD variations between populations (CEU vs. JPT+CHB, CEU vs. YRI, YRI vs. JPT+CHB) using genome positions from the Hapmap 3 reference. The red line represents the comparison between CEU (European ancestry) and JPT+CHB (East Asian ancestry), the purple line indicates CEU versus YRI (African ancestry), and the green line represents YRI versus JPT+CHB. Bottom Panel: Gene/transcript annotations are sourced from the reference downloaded from the UCSC database (EST, mRNA, uniGene, Encode, and RefGene). (TIF) [file pgen.1011151.s007.tif]

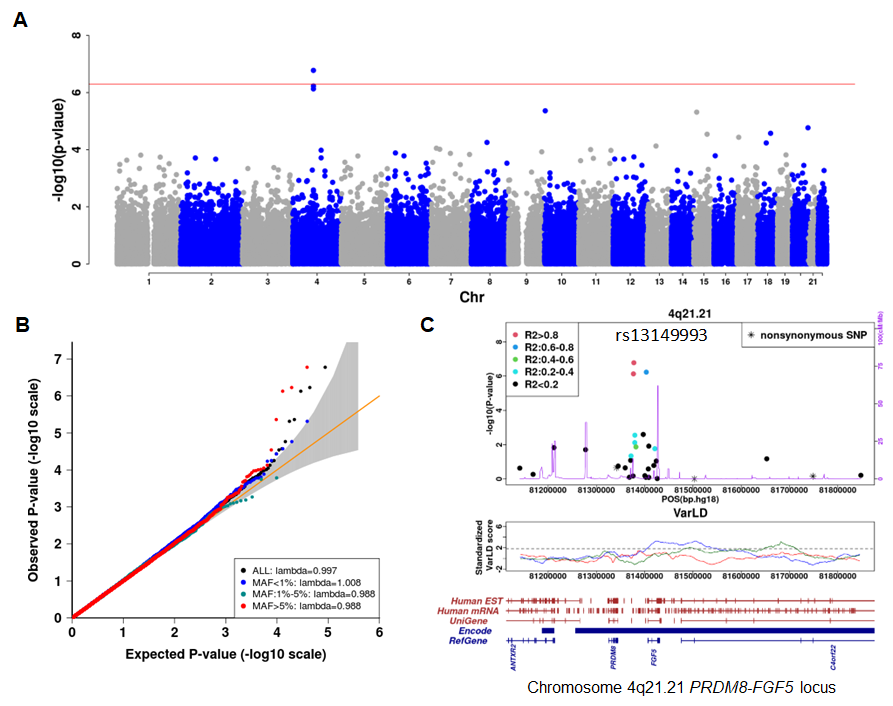

Supplement: S3 Fig — ExomeChip SNPs meeting quality control were analyzed in the EWAS. Results are shown in a (A) Manhattan plot and (B) QQ plot; the λGC value was 1.0. (C) Regional association plots with gene annotation for the chromosomes 4q21.21 region associated with pMAP is shown with the index SNP rs13149993 and additional SNPs within 500 kbp in each direction. Top panel: Regional pMAP association plot for the PRDM8-FGF5 locus. LD was calculated from our samples. Non-synonymous variants were annotated using ANNOVAR. The genetic recombination rate is based on Hapmap release 22. Middle Panel: Standardized varLD scores illustrate LD variations between populations (CEU vs. JPT+CHB, CEU vs. YRI, YRI vs. JPT+CHB) using genome positions from the Hapmap 3 reference. The red line represents the comparison between CEU (European ancestry) and JPT+CHB (East Asian ancestry), the purple line indicates CEU versus YRI (African ancestry), and the green line represents YRI versus JPT+CHB. Bottom Panel: Gene/transcript annotations are sourced from the reference downloaded from the UCSC database (EST, mRNA, uniGene, Encode, and RefGene). (TIF) [file pgen.1011151.s008.tif]

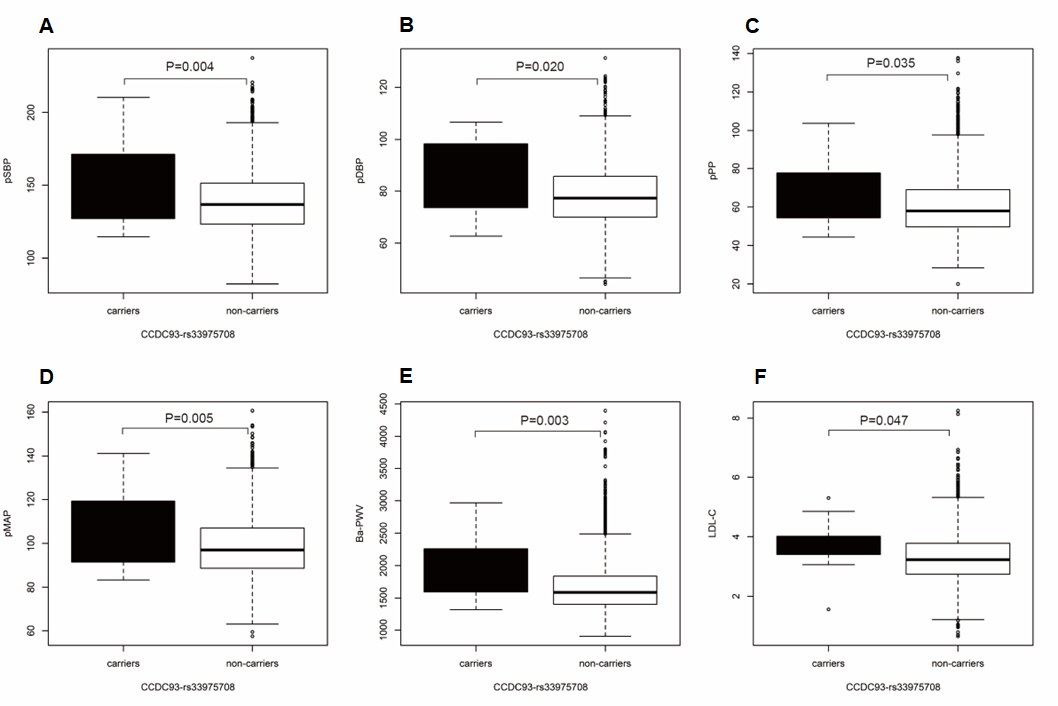

Supplement: S4 Fig — (A) Peripheral systolic blood pressure (pSBP); (B) Peripheral diastolic blood pressure (pDBP); (C) Peripheral pulse pressure (pPP); (D) Peripheral mean arterial pressure (pMAP); (E) Brachial-ankle pulse wave velocity (Ba-PWV); and (F) Low-density lipoprotein-cholesterol (LDL-C). All these traits were significantly higher in CCDC93 risk allele A carriers as compared to non-carriers. (TIF) [file pgen.1011151.s009.tif]

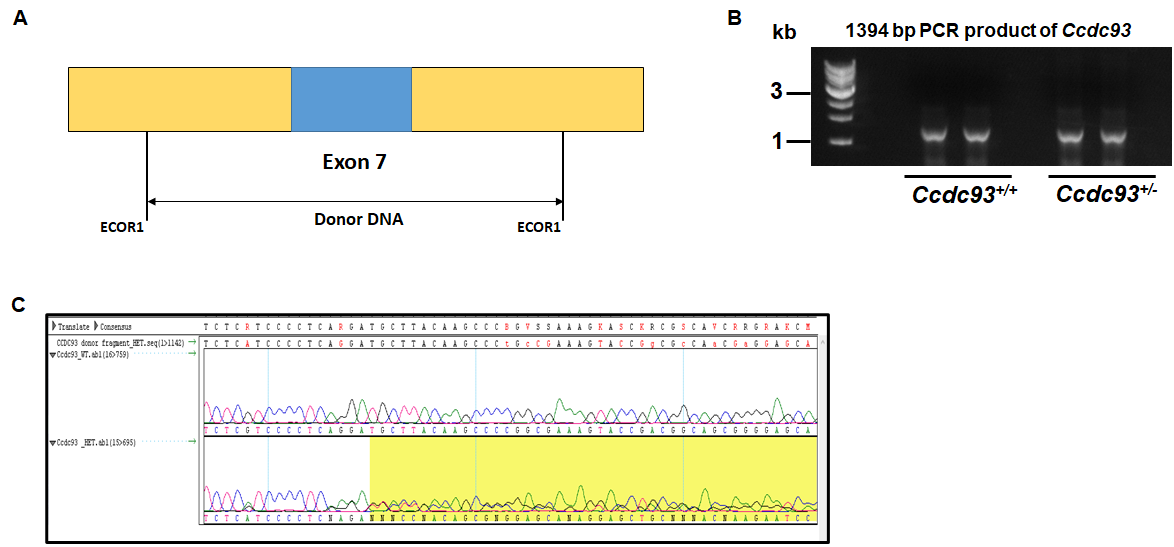

Supplement: S5 Fig — (A) Ccdc93 heterozygous mice were generated using the CRISPR/Cas9 system. (B) Ccdc93+/- carried a 21-nucleotide deletion. The molecular weight of DNA bands after PCR amplification of the Ccdc93 gene was not distinguishable by agarose gel electrophoresis. (C) Sanger sequencing of genomic DNA of Ccdc93 showed difference between littermate control and heterozygous of Ccdc93 with expected peak-on-peak DNA chromatogram observed in Ccdc93+/- at the transgenic site (yellow highlight). (TIF) [file pgen.1011151.s010.tif]

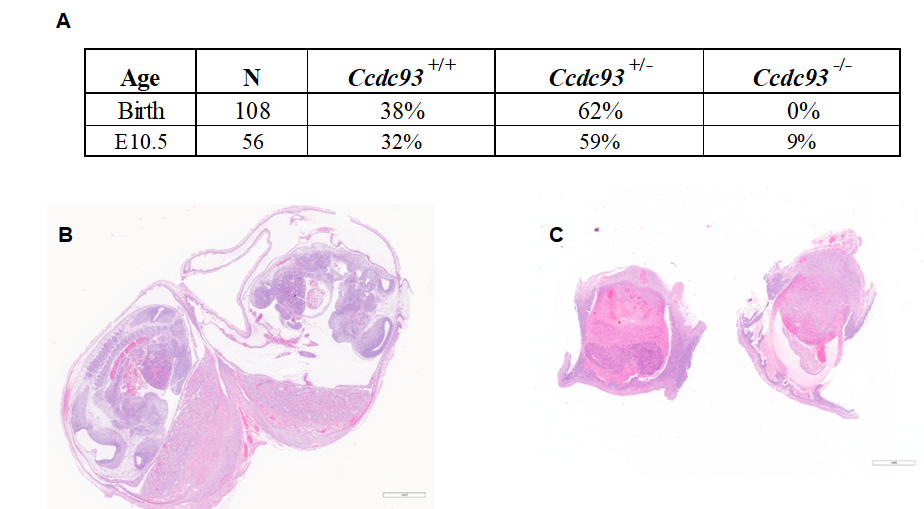

Supplement: S6 Fig — (A) Genotype frequencies at birth and at E10.5 of Ccdc93+/- X Ccdc93+/- mating. At birth, homozygous Ccdc93-/- mice were not viable and time mating at E10.5 of embryos produced from Ccdc93+/- X Ccdc93+/- mating showed Ccdc93 homozygosity were embryonic lethal and died before E10.5 of gestation. (B) Histology of fetoplacental units showed uterine horns from a Ccdc93+/- (heterozygous) pregnant female mouse at day E10.5 of gestation. This pregnancy was the product of Ccdc93+/- X Ccdc93+/- timed mating. Ccdc93+/- heterozygous embryos appeared viable (no evidence of necrosis, inflammation, or hemorrhage). (C) 2 resorption sites of Ccdc93-/- homozygous contained maternal hemorrhage and necrotic debris accompanied by neutrophilic and lymphocytic inflammation within and around chorioallantoic membranes on the antimesenterial (fetal) side. There were no embryos or embryo tissues present. (TIF) [file pgen.1011151.s011.tif]

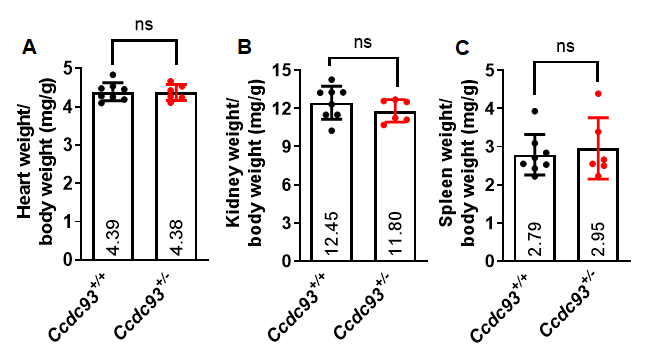

Supplement: S7 Fig — Tissue weight (A-heart weight, B-kidney weight, C-spleen weight) normalized over body weight were not different between the groups (N = 6–8 in each group). All data are shown as mean±s.d. (TIF) [file pgen.1011151.s012.tif]

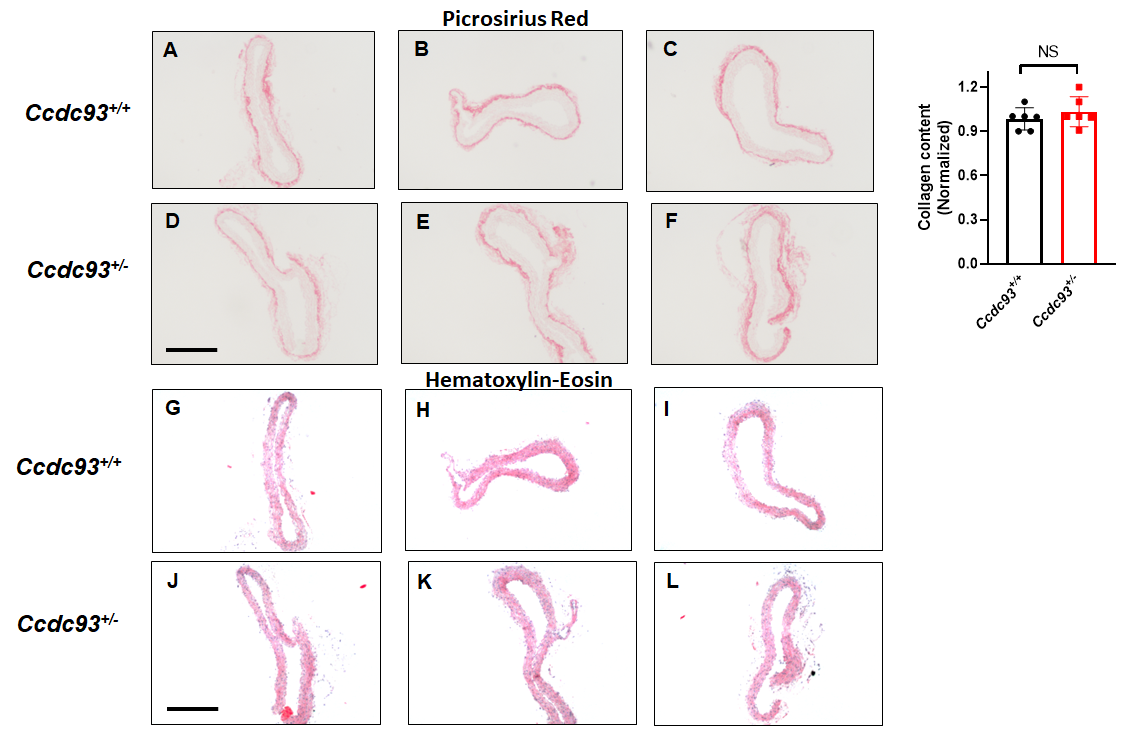

Supplement: S8 Fig — (A-C) Picrosirius red staining (PSR) for total collagen content of the descending thoracic aorta of littermate Ccdc93+/+ and (D-F) Ccdc93+/- mice demonstrate no significant differences. Hematoxylin and eosin (H&E) staining of the descending thoracic aorta of (G-I) littermate Ccdc93+/+ and (J-L) Ccdc93+/- mice did not demonstrate abnormal vascular morphology. Each section was examined under 4x objective microscope (Nikon’s Eclipse E600), photographed with a digital camera (DS-Ri1, Nikons Instrument), and evaluated by the ImageJ analysis system (NIH). PSR staining was used to facilitate automatic detection in pixel intensity by the image processing macro in ImageJ. N = 6 in each group. Scale bars 200 μm. All data are shown as mean±s.d. (TIF) [file pgen.1011151.s013.tif]

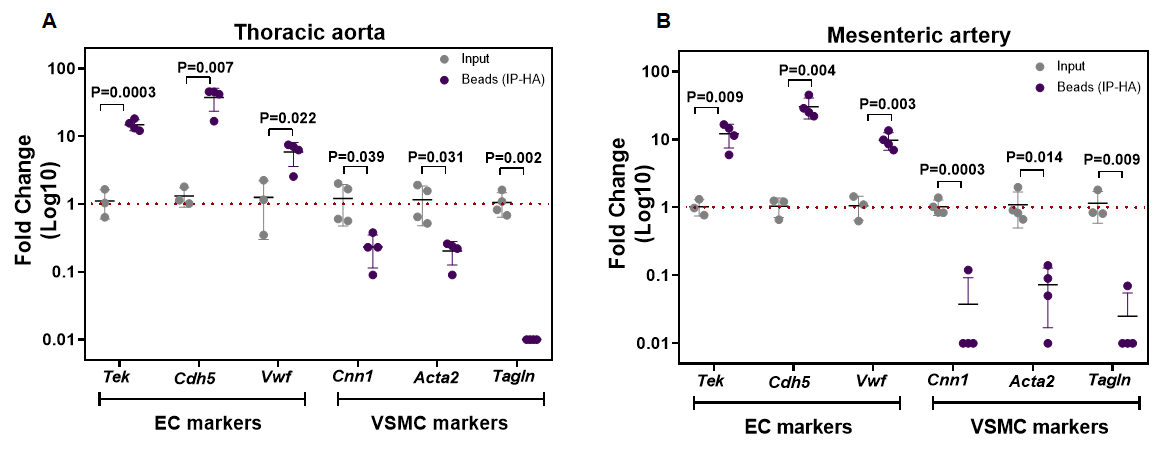

Supplement: S9 Fig — (A-B) Endothelial cell (EC)-specific enrichment and vascular smooth muscle-specific (VSMC) depletion of transcript markers by EC-TRAP in C57BL/6 wild-type mice in (A) thoracic aortae and (B) mesenteric arteries in vivo. Total RNA from was isolated from input and anti-HA beads (IP-HA) from descending thoracic aortae and mesenteric arteries of Rpl22fl/fl, Tie2-Cre+/0 (Tie2-RiboTag mouse) and showed expected enrichment of EC-specific transcripts (Tek, Cdh5 and Vwf) and depletion of vascular smooth muscle-specific markers (Cnn1, Acta2 and Tagln) in the IP-HA fraction as compared to input. Ccdc93 transcript expression in the Tek subpopulations (EC-specific) was enriched (Fig 2J), confirming predominantly EC expression. N = 3–4 in each group. All data are shown as mean±s.d. (TIF) [file pgen.1011151.s014.tif]

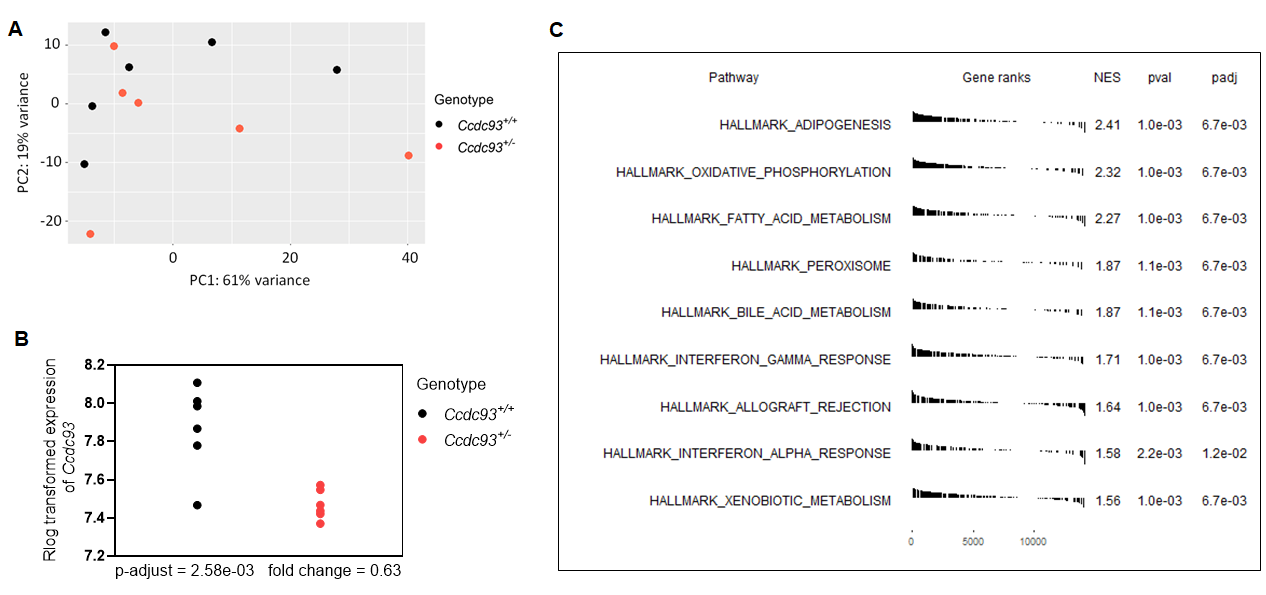

Supplement: S10 Fig — (A) Principal component analysis (PCA) plot showed no clear clustering by the genotype. The first and second principal components (PC1 and PC2) accounted for 61% and 19% variability in the RNA-Seq dataset. (B) Aortic Ccdc93 transcript expression was significantly reduced in Ccdc93+/- as compared to Ccdc93+/+ in bulk RNA-seq analysis, validating qPCR findings (Fig 2C). (C) Gene set enrichment analysis showed hallmark pathway associations with altered adipogenesis and fatty acid metabolism that were significantly upregulated in the thoracic aortae of Ccdc93+/- mice as compared to Ccdc93+/+ littermate controls, false discovery rate (FDR<0.1). (TIF) [file pgen.1011151.s015.tif]

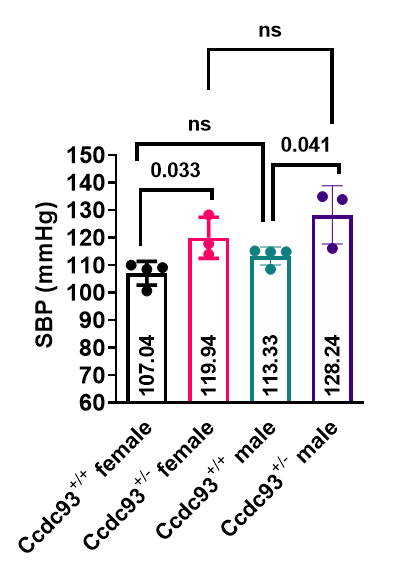

Supplement: S11 Fig — A similar increase in SBP was observed in male and female Ccdc93+/- heterozygous mice as compared to littermate controls. All data are shown as mean±s.d. (TIF) [file pgen.1011151.s016.tif]

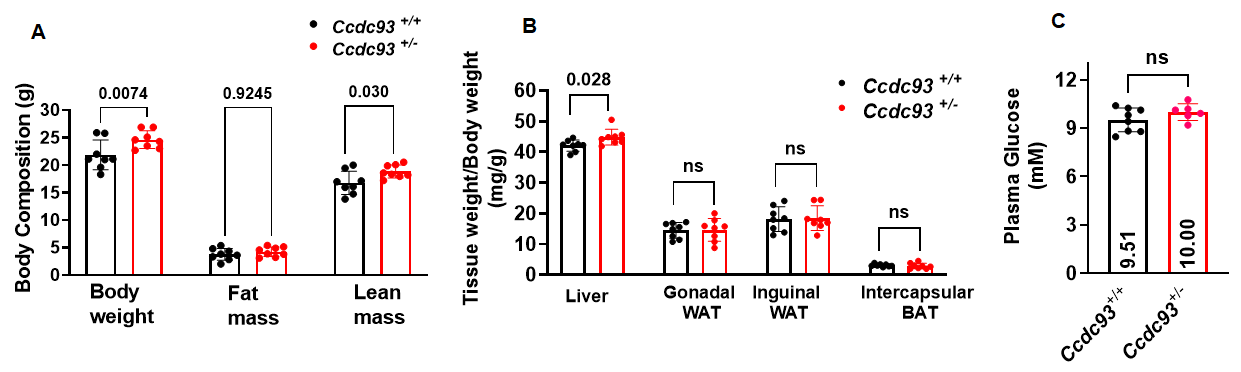

Supplement: S12 Fig — (A) Body weight, and lean mass measured by EchoMRI were significantly higher in Ccdc93+/- mice as compared to Ccdc93+/+ control mice. (B) A mild increase in the liver weight of Ccdc93+/- heterozygous mice was observed, however adipose tissue weight (gonadal and inguinal white adipose tissue, and interscapular brown adipose tissue) were not different between the genotype groups. (C) Plasma glucose level was equivalent in Ccdc93+/- heterozygous mice as compared to littermate control mice. Equal numbers of male and female mice (N = 4 per sex per genotype) were analyzed. White adipose tissue (WAT), brown adipose tissue (BAT). All data are shown as mean±s.d. (TIF) [file pgen.1011151.s017.tif]

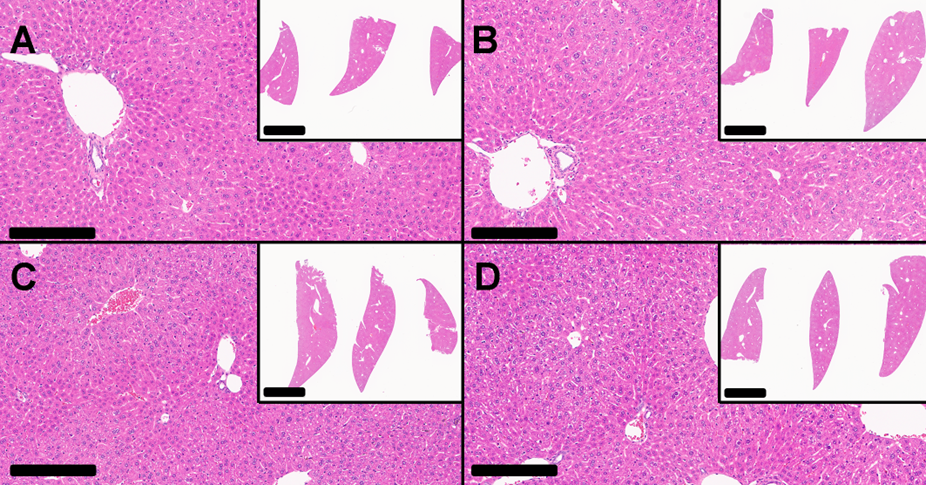

Supplement: S13 Fig — Hematoxylin and eosin stain (H&E) stained images are shown. No histological lesions were observed in the liver of Ccdc93+/- mice as compared to the littermate controls. (A) Male Ccdc93+/+ wild-type control liver samples (inset-male wild-type biological replicates, N = 3). (B) Male Ccdc93+/- heterozygous liver samples (inset-male Ccdc93+/- heterozygous biological replicates, N = 3). (C) Female Ccdc93+/+ wild-type control liver samples (inset-female wild-type biological replicates, N = 3). (D) Female Ccdc93+/- heterozygous liver samples (inset-female Ccdc93+/- heterozygous biological replicates, N = 3). Scale bars 200 μm, insets 2 mm. (TIF) [file pgen.1011151.s018.tif]

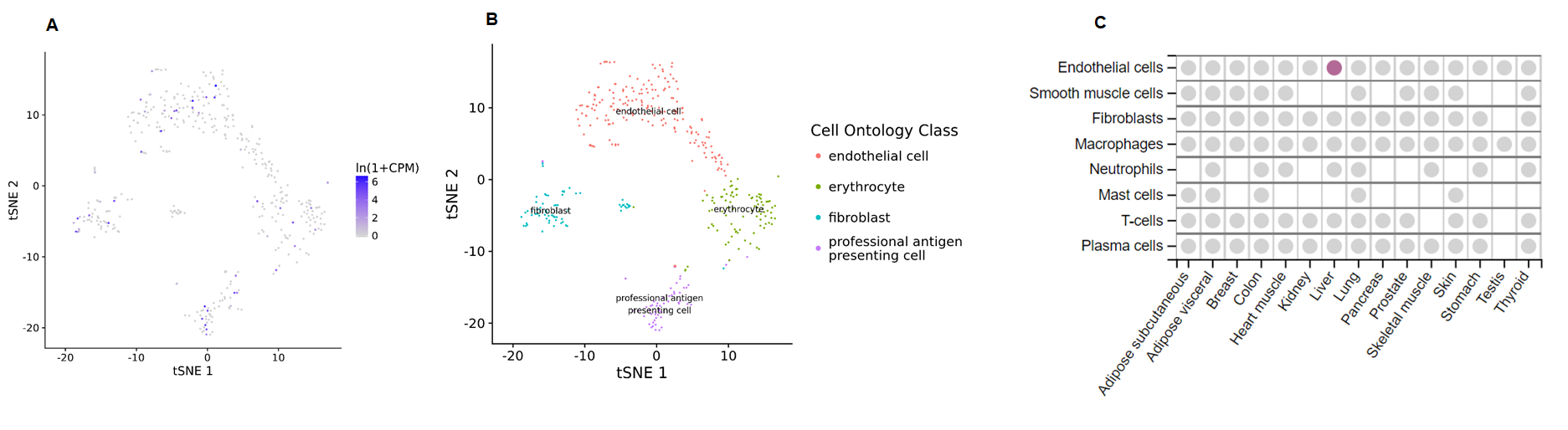

Supplement: S14 Fig — (A-B) Ccdc93 expression was queried in the mouse scRNA-seq database Tabula Muris (https://tabula-muris.ds.czbiohub.org/), and in (C) human scRNA-seq GTEx database protein atlas (https://www.proteinatlas.org/), which showed low tissue specificity of Ccdc93, and validated Ccdc93 expression in mouse aortic endothelial cells (blue dots in left figure panel A), shown in the tSNE plots, and enrichment of CCDC93 expression in human liver vascular endothelial cells (colored dot in panel C). (TIF) [file pgen.1011151.s019.tif]

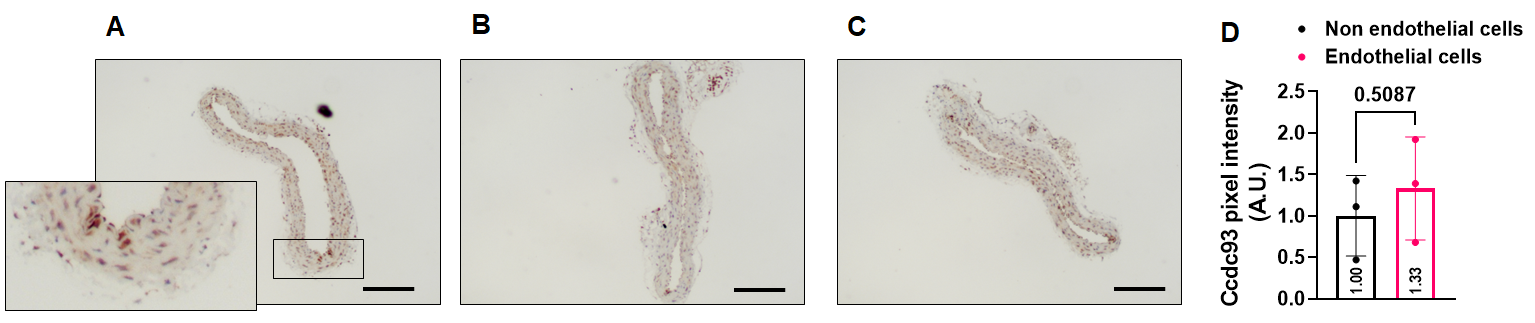

Supplement: S15 Fig — (A-C) Ccdc93 protein expression was observed in both endothelial and non-endothelial cells in wild-type mouse aortae (129/Sv). Three biological replicates of mouse thoracic aorta showed Ccdc93 expression (brown stained). (D) Pixel quantification showed 1.33-fold higher Ccdc93 protein expression in the endothelium (normalized by area) as compared to non-endothelial cells (P = NS). Scale bar = 200μm. (TIF) [file pgen.1011151.s020.tif]

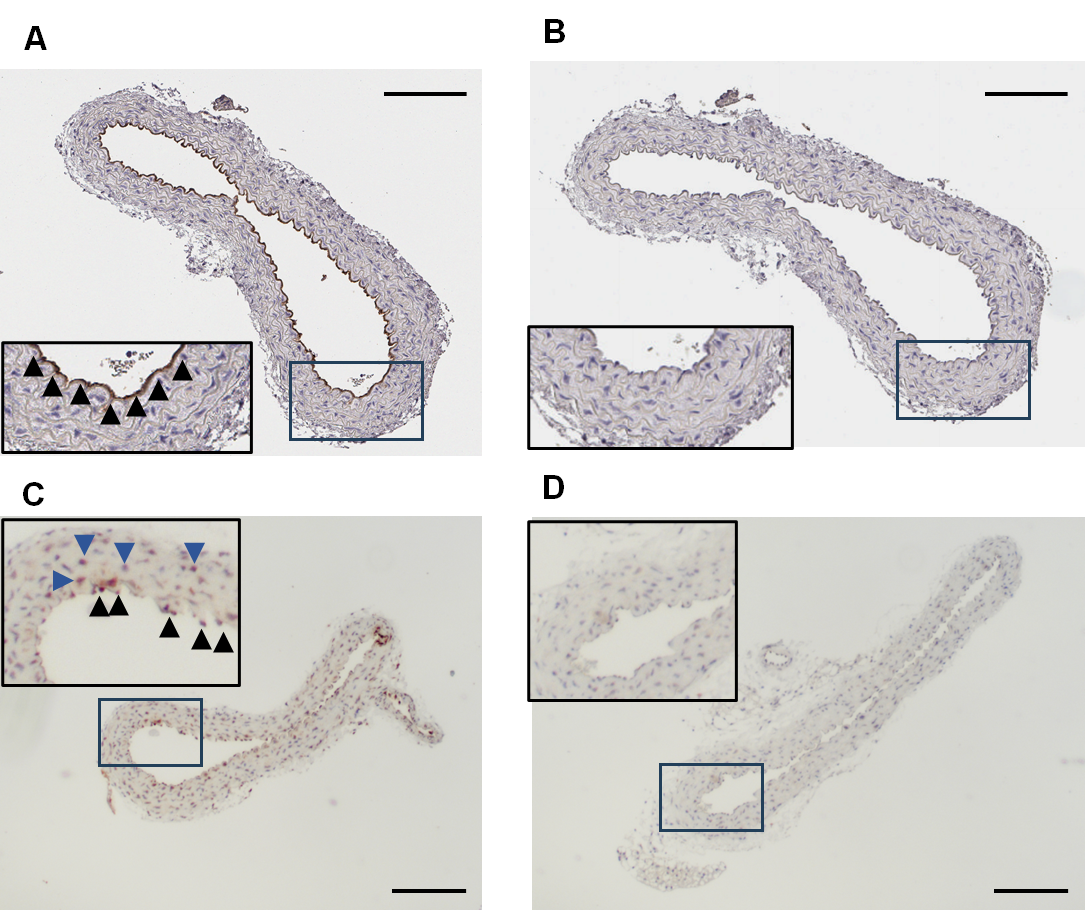

Supplement: S16 Fig — (A) Mouse aorta immunostaining of platelet and endothelial cell adhesion molecule-1 (PECAM-1), (B) Negative control (no antibody) immunostaining of mouse aorta serial section. (C) Positive control of mouse aorta immunostaining of Ccdc93. (D) Negative control of mouse aorta where Ccdc93 antibody was absent. Detection of PECAM-1 (black arrows) and Ccdc93 protein expression (black arrows represents EC expression and blue arrows represents medial expression) is based on horseradish peroxidase (HRP) catalysis of a 3,3′-Diaminobenzidine (DAB, brown) chromogenic reaction, with a hematoxylin (blue) nuclear counterstain, scale bar = 200μm. (TIF) [file pgen.1011151.s021.tif]
